# Large-scale brain connectivity changes following the administration of lysergic acid diethylamide, d-amphetamine, and 3,4-methylenedioxyamphetamine

**DOI:** 10.1038/s41380-024-02734-y

**Published:** 2024-09-11

**Authors:** Mihai Avram, Lydia Fortea, Lea Wollner, Ricarda Coenen, Alexandra Korda, Helena Rogg, Friederike Holze, Patrick Vizeli, Laura Ley, Joaquim Radua, Felix Müller, Matthias E. Liechti, Stefan Borgwardt

**Affiliations:** 1 Translational Psychiatry, Department of Psychiatry and Psychotherapy, University of Lübeck, Lübeck, Germany; 2 Center of Brain, Behavior and Metabolism (CBBM), University of Lübeck, Lübeck, Germany; 3 Institut dʼInvestigacions Biomèdiques August Pi i Sunyer (IDIBAPS), Barcelona, Spain; 4 Department of Medicine, University of Barcelona, Institute of Neuroscience, Barcelona, Spain; 5 Division of Clinical Pharmacology and Toxicology, Department of Clinical Research, University Hospital Basel, University of Basel, Basel, Switzerland; 6 Centro de Investigación Biomédica en Red de Salud Mental (CIBERSAM), Instituto de Salud Carlos III, Madrid, Spain; 7 Department of Psychiatry (UPK), University of Basel, Basel, Switzerland

**Keywords:** Neuroscience, Psychiatric disorders

## Abstract

Psychedelics have recently attracted significant attention for their potential to mitigate symptoms associated with various psychiatric disorders. However, the precise neurobiological mechanisms responsible for these effects remain incompletely understood. A valuable approach to gaining insights into the specific mechanisms of action involves comparing psychedelics with substances that have partially overlapping neurophysiological effects, i.e., modulating the same neurotransmitter systems. Imaging data were obtained from the clinical trial NCT03019822, which explored the acute effects of lysergic acid diethylamide (LSD), d-amphetamine, and 3,4-methylenedioxymethamphetamine (MDMA) in 28 healthy volunteers. The clinical trial employed a double-blind, placebo-controlled, crossover design. Herein, various resting-state connectivity measures were examined, including within-network connectivity (integrity), between-network connectivity (segregation), seed-based connectivity of resting-state networks, and global connectivity. Differences between placebo and the active conditions were assessed using repeated-measures ANOVA, followed by post-hoc pairwise t-tests. Changes in voxel-wise seed-based connectivity were correlated with serotonin 2 A receptor density maps. Compared to placebo, all substances reduced integrity in several networks, indicating both common and unique effects. While LSD uniquely reduced integrity in the default-mode network (DMN), the amphetamines, in contrast to our expectations, reduced integrity in more networks than LSD. However, LSD exhibited more pronounced segregation effects, characterized solely by decreases, in contrast to the amphetamines, which also induced increases. Across all substances, seed-based connectivity mostly increased between networks, with LSD demonstrating more pronounced effects than both amphetamines. Finally, while all substances decreased global connectivity in visual areas, compared to placebo, LSD specifically increased global connectivity in the basal ganglia and thalamus. These findings advance our understanding of the distinctive neurobiological effects of psychedelics, prompting further exploration of their therapeutic potential.

## Introduction

The use of psychedelic compounds, particularly substances like lysergic acid diethylamide (LSD), psilocybin, and N, N-dimethyltryptamine (DMT), has garnered significant attention due to the potential they have shown to reduce symptoms associated with various psychiatric disorders, such as depression, anxiety, and addiction [1–4]. Yet, despite their therapeutic promise, the precise mechanisms underlying psychedelic effects on the human brain remain incompletely understood. Comparing psychedelics with distinct substances having partly overlapping neurophysiological effects could shed some light on the complex mechanisms of action of these compounds.

Resting-state functional magnetic resonance imaging (rs-fMRI) has emerged as a powerful tool for probing large-scale connectivity patterns within the brain. In this study, we harnessed the capabilities of rs-fMRI to explore the impact of three distinct compounds: LSD, d-amphetamine, and 3,4-methylenedioxyamphetamine (MDMA) on brain connectivity patterns. Indeed, despite these compounds’ unique psychological effects and disparate actions on neurotransmitter systems, there are notable overlapping outcomes. LSD, which can induce profound alterations in visual and auditory perceptions, audiovisual synesthesia, derealization, and depersonalization, primarily exerts its influence as a partial agonist at the serotonergic 2 A receptor (5-HT2AR) [5, 6]. It also acts on other serotonergic receptors, as well as on dopaminergic and some adrenergic ones [7, 8]. MDMA is characterized by its capacity to enhance feelings of well-being and induce mild perceptual alterations, and relies predominantly on serotonergic neurotransmission [9–11]. However, MDMA also impacts dopaminergic neurotransmission and 5-HT2AR in its effects [7, 12]. d-Amphetamine, a compound with distinct psychological effects, including euphoria, changes in sexual desire, increased wakefulness, and improved cognitive control, elevates dopaminergic activity through its interactions with the dopamine transporter and dopamine release [13]. Overlapping effects between these substances (e.g., the feeling of connectedness [14, 15]) may arise from shared pharmacological pathways, for instance, via modulating dopaminergic neurotransmission. Alternatively, they could be a consequence of similar downstream effects resulting from interactions between the serotonergic, dopaminergic, and glutamatergic systems [16]. In line with these hypotheses, common neurophysiological effects of LSD, d-amphetamine, and MDMA have been noted for thalamocortical intrinsic functional connectivity (iFC) [17] and effective connectivity [18]. Furthermore, similar alterations in larger resting-state networks (RSNs) have also been observed between LSD and MDMA [19], although the two compounds have not yet been compared directly. Moreover, a more complete characterization of large-scale RSN changes, including in within-network (i.e., integrity) and between-network iFC (i.e., segregation), is missing for d-amphetamine. While such measures have been estimated for LSD and other psychedelics [20–24], direct comparisons with other substances are missing.

Our study aimed to investigate and compare the effects of LSD, d-amphetamine, and MDMA on three key aspects: (1) network integrity and segregation based on RSN templates, (2) network-dependent changes in whole-brain iFC, using a voxel-wise seed-based approach, and (3) global connectivity changes, assessed through degree centrality (DC). In line with previous findings, we hypothesized LSD to reduce the integrity of several RSNs compared to placebo with more pronounced effects in the default mode network (DMN) [21, 23, 25]. Based on previous qualitative comparisons [19], we expected MDMA to elicit similar changes in network integrity as LSD. However, we expected weaker effects for d-amphetamine, since it elicits less perceptual distortions and has a less complex neuropharmacological profile [7]. Similarly, we expected network segregation to be more reduced (i.e., increased between-network connectivity) for all substances compared to placebo across RSNs but more pronounced for LSD than MDMA – in line with previous findings for psilocybin [26] –, and respectively, d-amphetamine. We hypothesized similar changes for the voxel-wise seed-based analysis as for the network integrity and segregation analyses. Finally, we expected higher DC in the thalamus following LSD compared to the amphetamines, in line with our previous reports [17, 27].

In summary, our study aimed to discern the convergences and divergences in the modulation of large-scale connectivity patterns within the brain by these three compounds.

## Materials and methods

Data analyzed in this study were derived from the clinical trial NCT03019822 [7], conducted in Basel, Switzerland. The clinical trial received approval from the Ethics Committee for Northwest/Central Switzerland and the Federal Office of Public Health. All participants gave their written informed consent after receiving a comprehensive explanation of the study and were compensated financially for their involvement.

### Participants

This study collected data from 28 healthy participants, of which 25 were analyzed herein (12 female, mean age = 28.2 ± 4.35 years). Detailed information regarding the participants and experimental procedures can be found in the supplement and elsewhere [7, 17, 18]. The clinical trial used a double-blind, placebo-controlled, crossover design with four sessions involving the administration of 0.1 mg LSD, 40 mg d-amphetamine, 125 mg MDMA, and a placebo. The order of these sessions was randomized and counterbalanced among the participants.

Participants’ subjective experiences following substance administration were evaluated with the 11 dimensions of the Altered States of Consciousness Questionnaire (11D-ASC) [28] 11 h after substance administration.

Several physiological parameters (PPs), such as blood pressure, heart rate, and tympanic body temperature, were acquired immediately before (1.5 h after the administration of the substance) and after (2.5 h after substance administration) the fMRI scan [7]. The differences between active substances and placebo (∆PP) in average values of autonomic effects, before and after the fMRI scan, were employed in control analyses as in our previous approach [17].

### Data acquisition and preprocessing

We obtained structural and functional MRI data for all participants across conditions using a 3 T MRI system (Magnetom Prisma, Siemens Healthcare) equipped with a 20-channel phased array radio-frequency head coil. Imaging parameters can be found in the supplementary methods.

MRI data were preprocessed using the Configurable Pipeline for the Analysis of Connectomes (version 1.7.1., https://fcp-indi.github.io/) with the default preconfigured pipeline unless otherwise specified (https://fcp-indi.github.io/docs/v1.8.3/user/pipelines/preconfig). The preprocessing steps encompassed slice-timing correction, motion correction, scrubbing (FD Jenkinson, with a threshold of <0.2), intensity normalization, nuisance signal regression followed by bandpass filtering (0.01–0.1 Hz), registration to anatomical space, and normalization to a 3 mm isotropic voxel size in Montreal Neurological Institute (MNI) space using FSL FLIRT/FNIRT. The normalized images were smoothed using a 5 mm full width at half maximum isotropic Gaussian kernel. We employed nuisance signal regression using a unified multiple linear regression model, which integrated component-based noise correction (aCompCor) to mitigate physiological interferences [29]. Specifically, we retained 5 components each from the white matter (WM) and the cerebrospinal fluid (CSF) signal. Additionally, the influence of head motion was addressed through the Friston 24-parameter model, encompassing six head motion parameters estimated from the current volume, the corresponding six motion parameters estimated from the immediately preceding volume, and twelve corresponding squared terms. Considering the ongoing debate regarding the use of global signal regression (GSR) in psychedelic neuroimaging [30], we tested the effects of this denoising procedure and analyzed the data both with and without GSR.

### Resting-state network integrity and segregation analysis

The evaluation of RSN integrity (i.e., within-network iFC) and segregation (i.e., between-network iFC) was based on the seven-network parcellation from Yeo and colleagues [31]. We included the visual network (VIS), auditory-sensorimotor network (ASM), dorsal attention network (DAN), salience network (SAL), frontoparietal network (FPN), and the default-mode network (DMN). We excluded the limbic network (LIM) from further analyses due to its lower signal-to-noise ratio and reproducibility [32, 33]. To evaluate RSN integrity and segregation, we applied FSL’s dual regression [34]. This process involves two multivariate regressions for each subject. The first step regresses the RSN template from a subject’s 4D dataset, yielding a subject-specific time series. The second step regresses this time series from the same subject’s 4D dataset to obtain a subject-specific spatial map (parameter estimate (PE) images). The PE maps were used to assess RSN integrity. To achieve this, we computed the average PE across voxels within the investigated RSNs for each participant under every condition (LSD, d-amphetamine, MDMA, and placebo).

To conduct the segregation analysis, we used the time series derived from the initial step of the dual regression. Specifically, we calculated Pearson’s correlation coefficients for each pair of RSNs and then applied an r-to-z transformation to standardize these values. This procedure yielded a 6 × 6 matrix representing the iFC between the studied RSNs for each condition: LSD, d-amphetamine, MDMA, and placebo.

### Seed-based correlation analysis

We used C-PAC’s seed-based correlation analysis to investigate the iFC between the six RSNs and the whole brain. The Yeo-derived RSN templates served as seeds in this analysis. Seed-based iFC was computed between each network and every voxel in the brain, resulting in whole-brain z-maps for each network.

### Degree centrality

Degree centrality (DC) identifies the nodes with the most connections by counting the number of edges to all other nodes. Nodes with high DC will therefore have many direct connections with many other nodes in the network. In the C-PAC implementation, the voxel x voxel similarity matrix is calculated by the Pearson correlation between every pair of voxel time courses. Using the standard pipeline, only significant correlations were kept (P < 0.001) within the individual gray matter mask using AFNI’s 3dDegreeCentrality as implemented in C-PAC [35]. For each voxel, ‘weighted’ DC was computed as the average correlation coefficient across the remaining connections. Finally, a subject-specific DC map was generated and standardized using z-scores.

Similar measures of global connectivity have been used in past work with LSD [20, 27, 36], psilocybin [22, 36], and more recently DMT [24].

### Association between seed-based iFC and 5-HT2AR density

We investigated the potential relationship between RSN-derived seed-based iFC and 5-HT2AR density, derived from a single reference atlas [37]. We used the fslcc tool, part of the FSL package, to assess the overall similarity (cross-correlation) in spatial patterns between each RSN seed-based iFC map and a 5-HT2AR density map. The fslcc tool captures how well the spatial patterns of iFC correlate with the receptor density across subjects and conditions, providing a coefficient to summarize this relationship. Following this procedure, we obtained one correlation coefficient per subject, RSN, and condition (LSD, d-amphetamine, MDMA, and placebo). We then computed ANOVAs followed by post-hoc t-tests to assess differences between conditions. Multiple comparisons were corrected using the False Discovery Rate (FDR).

### Association between connectivity measures and subjective effects

We also explored potential relationships between RSN integrity, seed-based iFC, and global connectivity following the administration of every substance and subjective effect, as quantified by the subscales of the 11D-ASC with partial correlation analysis. Due to a large number of measures, we did not analyze associations with network segregation (i.e., several network pairs)—this measure is partly covered by seed-based iFC. Furthermore, we focused on seed-based iFC strength (i.e., increases vs. placebo) rather than decreases, which are partly covered by the network integrity findings (i.e., decreased within-network integrity). First, we identified the regions where seed-based iFC exhibited a significant increase in each condition compared to placebo. We then extracted iFC values from these regions reflecting increases for each experimental condition. Additionally, we also extracted values reflecting RSN integrity and DC changes. Finally, we investigated the relationship between a specific RSN/global connectivity and a specific dimension of the 11D-ASC, while controlling for ∆PP and differences in head motion (ΔFD).

### Statistical analysis

To investigate differences in network integrity and segregation across conditions, we conducted a repeated-measures analysis of variance (ANOVA), followed by post hoc pairwise t-tests for the RSNs and RSN pairs that exhibited statistical significance, respectively. All tests were two-tailed. We applied FDR correction for multiple comparisons in all statistical analyses. All reported P values are FDR-adjusted unless otherwise specified. Throughout the manuscript, FDR-adjusted *P* values are denoted as P_FDR_. The analyses were conducted in the software package R (version 4.2.2).

To investigate voxel-wise iFC and DC differences between conditions, we conducted one-way repeated-measures ANOVAs on the seed-based iFC maps of each template and the DC maps, respectively, using SPM12 (http://www.fil.ion.ucl.ac.uk/spm/). We defined a full factorial design with participants as the between-participant factor and conditions as the within-participant factor. Significant differences were based on a familywise error rate of *P* < 0.05 at the cluster level (*P* < 0.001).

## Results

The final participant sample included 25 healthy subjects. Neuroimaging data from these subjects has been analyzed previously [17, 18]. Notably, all the participants exhibited high-quality neuroimaging data, and no significant differences in head motion were observed between the sessions (mean FD, *F*_(3,72)_ = 1.80, *P* = 0.15).

### Network integrity and segregation

**Fig. 1:**
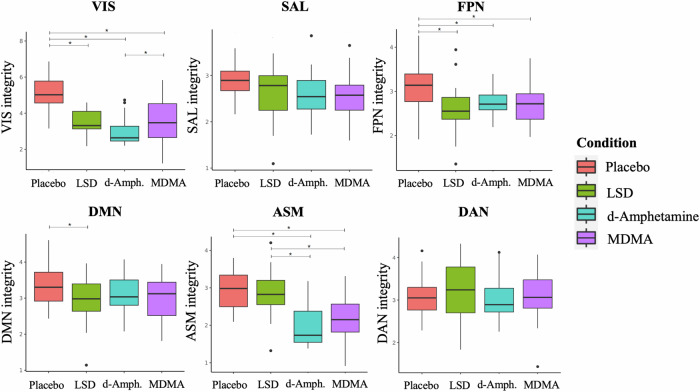
Substance-induced changes in network integrity. Depicted are changes in network integrity (i.e., within-network connectivity) induced by LSD, d-amphetamine, and MDMA, compared to placebo. All substances induced reductions in network integrity in the VIS and FPN. Amphetamines induced reduced integrity in SAL and ASM, whereas LSD uniquely reduced integrity in the DMN. Repeated-measures ANOVAs were computed on dual regression-derived parameter estimates and corrected for multiple comparisons (*P*_FDR_ < 0.05). The ANOVA was not significant for SAL and DAN. * - depicts significant differences. Abbreviations: VIS visual network, SAL salience network, FPN frontoparietal network, DMN default mode network, ASM auditory-sensorimotor network.

We found a statistically significant difference in network integrity across the four conditions after correction for multiple comparisons for the VIS (*F*_3,96_ = 31.3, *P*_FDR_ < 0.001), FPN (*F*_3,96_ = 7.7, *P*_FDR_ < 0.001), DMN (*F*_3,96_ = 3.3, *P*_FDR_ = 0.036), and ASM (*F*_3,96_ = 28.5, *P*_FDR_ < 0.001) (Fig. 1). Network integrity values are reported in Table S1. Post hoc analyses revealed a significant reduction in VIS integrity in all conditions compared to placebo. FPN integrity exhibited a significant reduction in all experimental conditions compared to placebo. DMN integrity was significantly reduced only for LSD compared to placebo. Finally, ASM integrity was decreased for d-amphetamine and MDMA compared to placebo. Regarding direct comparisons between the active substances, we found that d-amphetamine decreased integrity in VIS and ASM more than MDMA and LSD, respectively. Additionally, MDMA reduced integrity in the ASM more than LSD.

Including GSR in the analysis had noticeable effects on network integrity (Fig. S1). Briefly, GSR induced greater reductions in network integrity for all substances with stronger effects observed for LSD (see supplemental results for details).

**Fig. 2:**
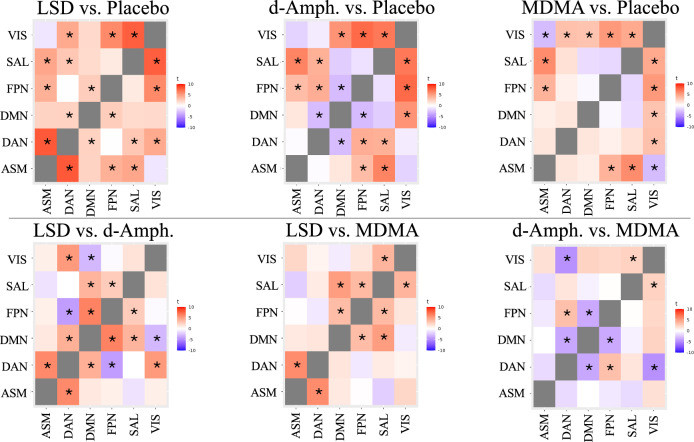
Substance-induced changes in network segregation. Depicted are changes in network segregation (i.e., between-network connectivity) induced by LSD, d-amphetamine, and MDMA, compared to placebo. LSD induced more extensive increases in connectivity. Compared to LSD, the amphetamines also induced increased segregation vs. placebo. Repeated-measures ANOVAs were computed on Pearson correlation coefficients of dual regression-derived time series of pairs of networks, followed by r-to-z transformations, and corrected for multiple comparisons (*P*_FDR_ < 0.05). * - depicts significant differences. Abbreviations: VIS visual network, SAL salience network, FPN frontoparietal network, DMN default mode network, ASM auditory-sensorimotor network.

In the network segregation analysis, the iFC between RSNs differed significantly across the four conditions except for the pair ASM and DMN (*F*_3,96_ = 2.5, *P*_FDR_ = 0.06) (Table S2, Fig. 2). When comparing all active substances to placebo, we consistently observed increased iFC between transmodal networks (DMN, SAL, FPN, DAN) and unimodal networks (ASM, VIS). LSD significantly increased between-network connectivity for almost all RSN pairs. In contrast, d-amphetamine reduced connectivity between the DMN and both DAN and FPN. Similarly, MDMA reduced the connectivity between the VIS and ASM.

Compared to d-amphetamine, LSD exhibited increased iFC between transmodal networks (DAN and DMN, FPN and DMN, SAL and DMN, SAL and FPN) with one exception (the connectivity between DAN and FPN was reduced). Similar findings were observed for the LSD vs. MDMA contrast, although less extensive (mainly for DAN). Finally, when comparing amphetamines, we observed that d-amphetamine exhibited increased iFC between DAN and FPN, but decreased iFC between DMN and FPN, DMN and DAN, and DAN and VIS compared to MDMA. For direct comparisons between the active substances see Table S3.

Including GSR as a preprocessing step significantly modified the results of the segregation analysis (Fig. S2). Briefly, GSR led to more extensive increases in segregation for all substances, again with stronger effects for LSD than for the amphetamines (see supplemental results for details).

### Seed-based correlation analysis

**Fig. 3:**
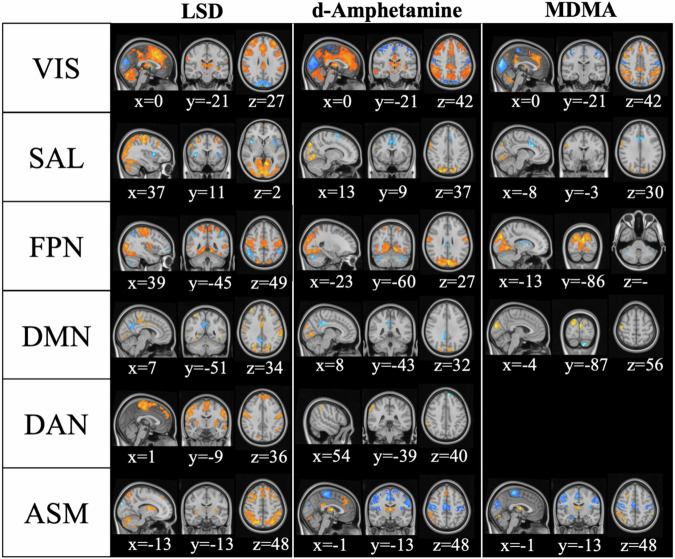
Substance-induced changes in seed-based connectivity. Depicted are voxel-wise repeated-measures analysis of variance (ANOVA) parametric maps reflecting contrasts between active substances and placebo for RSN whole-brain connectivity. The substances mainly increased connectivity between the seed network and other areas of the brain (shown in yellow/red) but reduced connectivity within the seed network (shown in blue). As no voxel-wise differences were observed between MDMA and placebo conditions for DAN iFC no image is depicted. The analyses were computed in SPM12 (*P* < 0.001, cluster-level familywise error–corrected *P* < 0.05); x, y, and z indicate Montreal Neurological Institute coordinates.

We investigated the voxel-wise iFC differences for each RSN with the whole brain across the four conditions (Table 1, Fig. 3). Briefly, when comparing all active substances with placebo, we observed that most RSNs showed increased iFC with regions belonging to other RSNs, particularly between unimodal and transmodal networks, with LSD showing the strongest effects. There was also decreased iFC within all RSNs except for DAN and ASM. However, in contrast to LSD, both amphetamines decreased iFC between the unimodal networks, VIS and ASM. For detailed results, see supplementary results and Fig. S3.

**Table 1:** Seed-based connectivity analysis: within- and between-network connectivity changes.

LSD vs*.* Placebo						
	VIS	SAL	FPN	DMN	DAN	ASM
VIS	**↓**	**↑**	**↑**	**↑**	**↑**	**↑**
SAL	**↑**	**↑ ↓**	**↑**	**↑**	**↑**	**↑**
FPN	**↑**	**↑**	**↓**	**↑**	**↑**	**↑**
DMN	**↑**	**↑**	**↑**	**↓**	**↑**	**↑**
DAN	**↑**	**↑**	**↑**	**↑**	**↑**	**↑**
ASM	**↑**					
d-Amphetamine vs*.* Placebo						
	VIS	SAL	FPN	DMN	DAN	ASM
VIS	**↓**	**↑**	**↑**	**↑**	**↑**	**↑ ↓**
SAL	**↑**	**↓**			**↑**	**↑ ↓**
FPN	**↑**		**↓**	**↓**	**↑**	**↑**
DMN	**↑**		**↓**	**↓**		**↑**
DAN			**↑**	**↓**		
ASM	**↓**	**↑**	**↑**	**↑**		**↓**
MDMA vs. Placebo						
	VIS	SAL	FPN	DMN	DAN	ASM
VIS	**↓**	**↑**	**↑**	**↑**	**↑**	**↓**
SAL	**↑**	**↓**			**↑**	**↑**
FPN	**↑**	**↓**	**↓**	**↓**	**↑**	**↑**
DMN	**↑**					**↑**
DAN						
ASM	**↓**	**↑**	**↑**	**↑**	**↑**	**↓**
LSD vs. d-Amphetamine						
	VIS	SAL	FPN	DMN	DAN	ASM
VIS	**↑**	**↑**			**↑**	**↑**
SAL	**↑**	**↑**	**↑**	**↑**	**↑**	**↑**
FPN		**↑**	**↑**	**↑↓**	**↓**	**↑**
DMN		**↑**	**↑**	**↑**	**↑**	**↑**
DAN	**↑**	**↑**	**↑↓**	**↑**	**↑**	**↑**
ASM	**↑**	**↑**	**↑**	**↑**	**↑**	**↑**
LSD vs*.* MDMA						
	VIS	SAL	FPN	DMN	DAN	ASM
VIS		**↑**	**↑**	**↑**	**↑**	**↑**
SAL	**↑**	**↑**	**↑**	**↑**	**↑**	**↑**
FPN	**↑**	**↑**	**↑↓**	**↑**		**↑**
DMN		**↑**	**↑**	**↑**		**↑**
DAN	**↑**	**↑**	**↑**	**↑**	**↑**	**↑**
ASM	**↑**		**↑**	**↑**	**↑**	**↑**
d-Amphetamine vs. MDMA						
	VIS	SAL	FPN	DMN	DAN	ASM
VIS						
SAL						
FPN						
DMN					**↓**	
DAN	**↓**			**↓**		
ASM						**↓**

Depicted are increases (arrows pointing up) and decreases (arrows pointing down) between the iFC of a seed network and regions belonging to other networks. Overlaps were calculated between voxels demonstrating changes in iFC (cluster > 20) and the other RSN templates. Only significant effects are shown.

Including GSR as a preprocessing step significantly modified the results of the seed-based iFC analyses (see Figs. S4, S5, and supplemental results for details). While the ‘direction’ of the results—i.e., increased between RSN iFC and decreased iFC within the seed-RSN—remained mainly consistent, we observed an overall decrease in between-network connectivity with more extensive effects for LSD compared to d-amphetamine and MDMA.

### Degree centrality

**Fig. 4:**
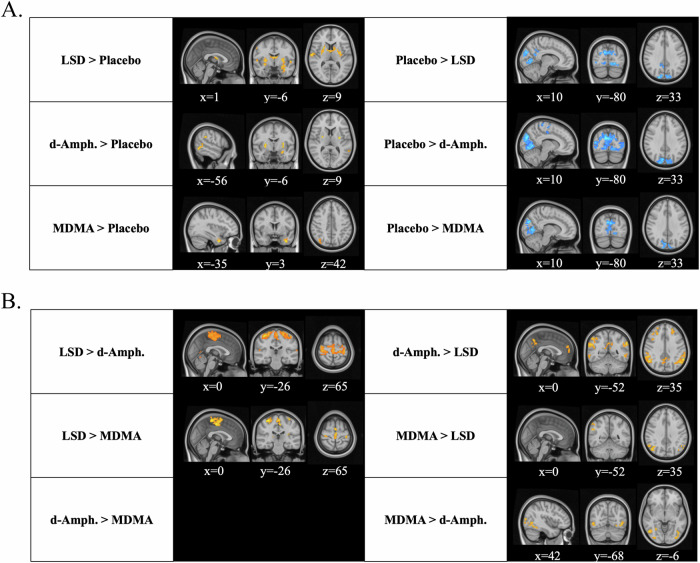
Substance-induced changes in degree centrality. Depicted are voxel-wise repeated-measures analysis of variance (ANOVA) parametric maps reflecting contrasts between active substances and placebo for global connectivity (**A**) and between the active conditions only (**B**). Compared to placebo, substances differentially increased global connectivity (i.e., degree centrality) in the brain (shown in yellow/red) but similarly induced reductions in visual areas (shown in blue). Compared to the amphetamines directly, LSD mainly increased global connectivity in sensorimotor areas. As no voxel-wise differences were observed between d-amphetamine and MDMA, no image is depicted. The analyses were computed in SPM12 (*P* < 0.001, cluster-level familywise error–corrected *P* < 0.05); x, y, and z indicate Montreal Neurological Institute coordinates.

We computed degree centrality (DC) and compared across conditions to investigate global connectivity changes induced by the active substances (Fig. 4). Similar global connectivity approaches have been used to investigate the effects of several serotonergic psychedelics, including LSD [20, 27, 36]. Compared to placebo, LSD demonstrated higher DC in the basal ganglia and thalamus as well as in areas overlapping parts of the ASM and the SAL, while concurrently exhibiting lower DC in VIS areas. d-Amphetamine induced higher DC in the basal ganglia and areas overlapping the FPN and SAL. We also observed lower DC in the VIS and ASM areas, specifically the paracentral gyrus and right precentral gyrus. MDMA elicited higher DC in the left temporal pole and parts of FPN, but lower DC in VIS and ASM, akin to d-amphetamine.

Furthermore, LSD increased DC in areas overlapping ASM, such as the right superior temporal gyrus and right precentral gyrus, compared to both amphetamines. However, LSD showed reduced DC in the right angular gyrus, overlapping the DMN, compared to both amphetamines. Areas overlapping the FPN, such as the frontal gyrus, left angular gyrus, and bilateral supramarginal gyrus, also showed decreased DC for LSD compared to d-amphetamine. Similarly, parts of the DMN were also reduced for LSD compared to d-amphetamine. d-Amphetamine elicited decreased DC in the VIS compared to MDMA.

Including GSR as a preprocessing step slightly modified the results of the global connectivity analysis (see Fig. S6 and supplemental results for details). Unlike the other analyses, the findings remained relatively consistent for DC.

### Control analyses for head motion and physiological parameters

We tested whether our measures of interest were associated with head motion and physiological parameters (PP) with Pearson’s correlation analysis. Specifically, we investigated whether substance-induced changes (i.e., substance - placebo) in integrity, seed-based iFC, and DC were associated with ∆FD and ∆PP. With one exception (temperature and reduced DC for LSD), none of the observed correlations between substance-induced changes and ∆PP and ∆FD would survive correction for multiple comparisons. Some LSD-induced changes in network integrity and seed-based iFC correlated with certain ∆PP. d-Amphetamine- and MDMA-induced changes did not correlate with ∆PP, but there were associations with ∆FD. For details, see Table S4.

### Association between seed-based iFC and 5-HT2AR density

**Fig. 5:**
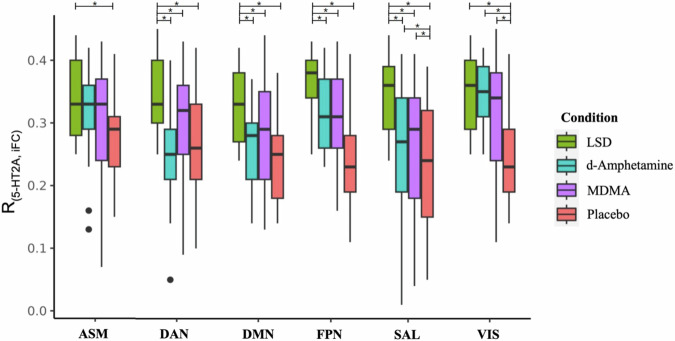
Associations between altered connectivity and 5-HT2AR density. Depicted are comparisons of Pearson’s correlation coefficients between the iFC of RSNs and 5-HT2AR density in the same regions for all conditions. Notably, LSD showed a stronger positive association with the 5-HT2AR density across all networks, except for VIS, compared to both placebo and amphetamines. Whiskers represents the minimum and maximum correlation coefficients values within 1.5 times the interquartile range. Repeated-measures ANOVAs were computed on the Pearson coefficients and corrected for multiple comparisons (P_FDR_ < 0.05). * - depicts significant differences. Abbreviations: VIS visual network, SAL salience network, FPN frontoparietal network, DMN default mode network, ASM auditory-sensorimotor network.

The analysis regarding the association between specific RSN seed-based iFC and overlapping 5-HT2AR density showed stronger positive associations for LSD than for placebo and both amphetamines, apart from ASM and VIS (Fig. 5). In addition, both amphetamines also showed stronger positive associations between 5-HT2AR density and the seed-based iFC of SAL and VIS, respectively, compared to placebo.

### Association between connectivity measures and subjective effects

We used partial correlation analyses to explore the relationships between the investigated connectivity metrics and subjective effects evaluated by 11D-ASC while controlling for physiological parameters and head motion. For details, see Table S5. Briefly, for LSD, only network integrity and decreased DC correlated with some subjective effects. DMN integrity was associated with the most subjective effects. For d-amphetamine, only network integrity correlated with subjective effects, most of these being related to SAL. For MDMA, network integrity of the DMN and ASM correlated with some subjective effects. Similar findings were observed for the 5D-ASC, see supplemental results and Table S6.

## Discussion

In this study, we employed pharmacological fMRI to investigate the shared and distinct effects of LSD, d-amphetamine, and MDMA on various large-scale connectivity measures. These included network integrity (within-network iFC), network segregation (between-network iFC), voxel-wise seed-based iFC of distinct RSNs, and global connectivity (degree centrality; DC). Additionally, we explored the associations between functional connectivity changes and 5-HT2AR density, as well as subjective effects. Our findings revealed both commonalities and unique alterations induced by the three substances. Notably, compared to the amphetamines, LSD led to (i) decreased integrity in the DMN, (ii) more extensive increases in between-network iFC, (iii) broader connectivity increases between most of the investigated RSNs and other brain regions, (iv) stronger associations between voxel-wise RSN iFC and 5-HT2AR density, and (v) increased DC in the basal ganglia and thalamus. These results underscore the distinctive neurobiological effects of LSD, shedding light on the unique alterations induced by this psychedelic compound.

### Distinct effects of LSD

The present study sheds new light on the effects of classic psychedelics compared to other mind-altering substances. Notably, we found that LSD had unique effects on distinct connectivity measures, compared both to d-amphetamine and the ‘atypical psychedelic’ MDMA. We observed that only LSD reduced the network integrity in the DMN, compared to placebo. This finding is in line with several previous reports of psychedelic-induced DMN integrity decreases [21, 24, 25, 38, 39]. This reduction in integrity showed a trend association with an increased feeling of “experience of unity” and “oceanic boundlessness”, which is also consistent with previous findings concerning psychedelic phenomena [39, 40]. These observations suggest that alterations in DMN integrity may underlie some of the unique subjective experiences associated with LSD.

LSD induced more extensive changes in between-network connectivity compared to the amphetamines, leading to a general increase in connectivity between transmodal networks (e.g., DMN, FPN) and between these and unimodal networks (e.g., ASM, VIS). This increase potentially underlies the breakdown of conventional cognitive and perceptual boundaries, contributing to the characteristic psychedelic experience [36]. Notably, no decreases in between-network connectivity were observed for LSD. While not all effects were unique, direct comparisons between LSD and d-amphetamine and MDMA, respectively, demonstrated that connectivity between the DMN, FPN, and SAL was stronger for LSD than for the amphetamines. This pattern was also observed in voxel-wise seed-based iFC analyses, where LSD induced greater connectivity between the RSNs covering transmodal cortices (SAL, FPN, DMN, and DAN) than both placebo and the amphetamines. Increases in between-network iFC are among the most frequently reported imaging findings for psychedelics [21, 24, 26, 36] and have been suggested as a relevant mechanism behind the therapeutic effects of these compounds [41]. The transmodal cortices covered by the DMN, FPN, DAN, and SAL also contain the highest density of 5-HT2A receptors [37, 42]. Therefore, it is not surprising that the associations between these networks’ iFC and 5-HT2AR density were stronger for LSD than for the amphetamines and placebo. These findings are consistent with recent reports for psilocybin, LSD, and N,N-dimethyltryptamine (DMT), emphasizing the link between connectivity changes and 5-HT2AR [24, 43].

We also observed stronger connectivity of the sensorimotor cortices following LSD compared to the other conditions. Interestingly, beyond iFC, we also found increased global connectivity (i.e., degree centrality – DC) in these areas but also in the basal ganglia, and the thalamus following LSD compared to placebo. Psychedelic-induced increased global connectivity in the basal ganglia and thalamus has been linked to the thalamic filter model [22, 27, 36]. This model suggests that psychedelics impair the filter function of the thalamus, thereby leading to an information overflow to the cortex, which is in turn linked to psychedelic phenomena [44–46]. While psychedelic-induced changes in global connectivity have been investigated with various methods, leading to somewhat inconsistent results in the literature (see supplementary discussion and Table S7), increased global connectivity in the basal ganglia and thalamus is the most consistent finding across the various studies, independent of the used methodology [20, 27, 36]. Surprisingly, although seed-based hyperconnectivity between the ASM and the thalamus was observed for all substances (Fig. 3) and reported previously [17, 18], thalamic DC was not increased for the amphetamines compared to placebo, suggesting that the thalamus may play a more central role in the overall brain network for psychedelics than amphetamines.

Together, these findings indicate that several of the consistently reported psychedelic-induced changes in neuroimaging metrics (e.g., alterations in DMN, increased between-network connectivity, and global connectivity in the basal ganglia and thalamus) may be unique to serotonergic psychedelics and are not induced (or less extensively) by other mind-altering compounds with partly overlapping neuropharmacological profiles. This is highly relevant, as it informs on psychedelic-specific targets for therapeutic action. Notably, these effects have mostly been reported in acute states (for exceptions see [41, 47, 48]) and it is unclear how they relate to presumably slower-acting neuroplastic processes, previously reported for psychedelics [49, 50]. Future studies need to clarify this putative link.

### Comparison between d-amphetamine and MDMA

d-Amphetamine and MDMA elicited similar changes across several investigated metrics. For instance, their effects on network integrity were nearly identical with notable specific decreases observed in the ASM and SAL, consistent with previous findings [19, 51]. Furthermore, both substances increased between-network connectivity, primarily between the VIS and several transmodal networks. Additionally, nearly identical patterns were observed for the SAL and ASM in the seed-based voxel-wise analysis. These similarities are intriguing given the mainly distinct pharmacological profiles and neurotransmitter systems influenced by each substance—predominantly dopaminergic for d-amphetamine and serotonergic for MDMA [7]. However, both compounds are structurally related and stimulate norepinephrine release [52], possibly leading to similar pharmacodynamic properties, resulting in more aligned fMRI-derived functional outcomes. The structural similarity or norepinephrinergic effects may facilitate comparable interactions with certain neural circuits, especially those involved in sensory processing and cognitive control. Additionally, interactions between dopaminergic and serotonergic neurotransmitter systems [16] may also contribute to the similar effects observed on distinct functional connectivity metrics.

Nevertheless, notable differences were also observed between the amphetamines. For instance, the more pronounced effects of d-amphetamine on the VIS may be related to amphetamine’s stronger dopaminergic effects. Dopamine plays an important role in modulating visual processing and attention [53]. In support, the connectivity between VIS and SAL was also increased for d-amphetamine in comparison to MDMA, suggesting increased salience for visual processing. Furthermore, d-amphetamine exhibited greater connectivity between attentional (DAN) and cognitive/executive (FPN) networks, which aligns with its subjective effects on enhanced cognitive control [13]. Additionally, more extensive network segregation was observed for d-amphetamine compared to MDMA, possibly related to amphetamine-induced increased focus [54]. Another notable difference was the distinct effect the compounds had on the interaction between transmodal networks. Compared to MDMA, d-amphetamine increased segregation in these networks, particularly for the DMN. Conversely, MDMA elicited effects more similar to LSD on some core transmodal networks, such as increased DMN-FPN connectivity, when compared to d-amphetamine directly. Remarkably, however, across the analyses, MDMA’s effects more closely matched those of d-amphetamine than LSD. This was an unexpected result based on previous reports, which suggested rather similar connectivity alterations between psychedelics and MDMA [19]. Nevertheless, the direct comparisons between the compounds conducted herein, clearly demonstrate that despite some similarities, MDMA’s effects markedly differ from LSD’s on several network-based and whole-brain-derived neuroimaging metrics. While some of the subjective and therapeutic effects of MDMA may overlap with classic psychedelics like LSD [7, 19, 55], its neural impact appears to align more closely with stimulants such as d-amphetamine, challenging the classification of MDMA as an ‘atypical psychedelic’. This intermediate positioning highlights the complexity of MDMA’s action on the brain, a substance that leverages mechanisms from both stimulants and psychedelics. Understanding this dual influence can inform both the clinical application and the development of new therapeutic strategies that harness these combined effects. Additionally, it would be valuable to investigate how MDMA compares to other stimulants (e.g., methylphenidate) in terms of its effects on brain connectivity and function. Such comparisons could help elucidate the specific neural circuits and mechanisms that underlie the effects of MDMA, further informing both clinical practice and drug development. On a similar note, comparison studies may also illuminate whether the perceived similarities in effects are mirrored at the neural level for distinct psychedelics, such as LSD, DMT, and psilocybin, which may also exert some unique influences on fMRI-derived functional parameters (i.e., as seen for MDMA and LSD here). Such investigations hold significant clinical relevance, especially considering the ongoing exploration of distinct psychedelics for various psychiatric disorders, such as LSD for anxiety disorders [3] and psilocybin for depression [56].

### Common changes across mechanistically distinct substances

Compared to placebo, all substances reduced network integrity in the VIS and FPN, indicating shared neurophysiological effects. Decreased integrity in the VIS has been previously reported for several psychedelics [21, 24, 25] and MDMA [19]. To the best of our knowledge, amphetamine-induced changes in within-VIS iFC have not yet been reported; however, there is some evidence of within-VIS iFC decreases after methamphetamine [57]. Interestingly, the lower integrity found in VIS was paralleled by an increase in between-network connectivity between the VIS and several other networks, including the DMN, FPN, and SAL, for all substances compared to placebo. This increased between-network connectivity suggests that while the VIS becomes less modular, it integrates more with other networks, potentially leading to altered sensory and cognitive experiences [25]. Additionally, we observed a decrease in DC in areas covering VIS for all substances against placebo. Reduced DC suggests less correlated activity between the visual system and the rest of the brain. This finding may be a corollary of the reduced integrity, indicating local network-based effects rather than global changes per se. Alternatively, a reduction in DC may reflect that the VIS is less central within the overall brain network following substance administration. Put differently, despite substance-induced increased between-network connectivity, the overall number of connections involving the VIS may be reduced, or its connections may be less influential in the global network structure compared to other regions.

While integrity decreases have been reported for the FPN following psychedelics [24, 25], our findings are somewhat discrepant for d-amphetamine [51] and MDMA [19]. We note, however, that distinct methods have been utilized to compute integrity, including average connection strength between regions-of-interest (ROIs) comprising a network [22], voxel-wise approaches [19], and dual regression approaches [24], as computed here. We speculate that some discrepancies between studies may be driven by such methodological differences. Additionally, the MDMA condition from the present study was included together with another independent cohort in Müller and colleagues’ MDMA study [19]. Discrepancies vis-à-vis this study may, therefore, also reflect a difference in sample size.

The shared effects on VIS are intriguing given the mechanistically distinct actions of LSD, d-amphetamine, and MDMA. One potential explanation for the common drug effects may be related to the shared dopaminergic properties [7]. As mentioned above, dopamine plays a significant role in visual processing, and increased dopaminergic activity can enhance neural activity and connectivity in regions associated with visual perception [58]. We observed increased connectivity between VIS and other areas and concurrently regional decreases. Our speculation is also supported by findings that methylphenidate-induced striatal dopamine increases are associated with concurrent decreases in the amplitude of low-frequency fluctuation (ALFF; a metric used to detect the regional intensity of spontaneous fluctuations in the BOLD signal [59]), particularly in the primary visual cortex [60].

Overall, our findings suggest that despite the predominately distinct pharmacological profiles of LSD, d-amphetamine, and MDMA, they share some common effects on brain connectivity. In turn, these common effects may be linked to common pharmacological actions related to the modulation of sensory processing and some cognitive networks. These findings highlight the importance of considering not just the primary pharmacological actions of a drug, but also its broader impact on brain connectivity. This perspective can lead to a more comprehensive understanding of how different substances interact with the brain, potentially revealing new avenues for pharmacological research and therapy.

### Limitations

Our investigation is not without limitations. First, due to the modest sample size, the study may have been underpowered to detect certain effects. For instance, by combining the MDMA condition from this study’s participants with that of another independent study (total n = 52) Müller and colleagues [19] reported somewhat discrepant effects. For instance, the DC findings appear coherent, but are more extensive in the Müller study, with increases in several sensorimotor areas. Furthermore, while voxel-wise iFC changes for unimodal networks are consistent with the present study, Müller and colleagues also reported iFC decreases within-DMN and increases within-FPN, not seen herein. Besides differences in sample size, slight methodological variability (e.g., networks derived from ICA vs. templates, etc.) may also account for the inconsistent findings. Second, we cannot exclude that changes in some physiological parameters and head motion did not bias the connectivity measures after substance administration. While the link between connectivity measures and physiological parameters and motion was weak and not significant (excepting temperature for LSD-related DC) we caution the reader about possible effects. Third, we found that including GSR in preprocessing leads to considerable changes across all analyses. There is an ongoing debate in the psychedelic neuroimaging community regarding the inclusion of GSR as standard practice [30]. Based on our findings, we caution against the routine use of GSR in psychedelic neuroimaging studies. The significant and differential effects observed across all analyses and within-condition comparisons (see Fig. S7) suggest that its application could obscure true neural signals and potentially introduce biases, particularly in studies involving substances with distinct physiological and psychological effects (see supplemental discussion for details). Fourth, the doses of LSD, d-amphetamine, and MDMA were not calibrated to ensure equivalent pharmacological efficacy across substances. It is, therefore, possible that the observed differences between substances do not necessarily indicate intrinsic differences in drug effects but also differences in dose. Future studies may consider dose-response in more detail to elucidate the relative contributions of dose versus drug-specific effects on brain connectivity measures. Fifth, concerning the associations between RSN seed-based iFC and 5-HT2AR, we acknowledge that pooling data from separate cohorts—the iFC data from our study and receptor density data from the PET atlas—limits the ability to directly correlate individual differences in connectivity with receptor levels. Future research integrating simultaneous PET-fMRI would be needed to address this limitation and provide a more precise understanding of the relationship between functional connectivity and receptor density. Probably the most serious drawback of our study lies in the possible carry-over effects. Recent research has shown that the effects of psychedelics on connectivity measures can last for several weeks, if not longer [41, 47, 48]. Since condition crossover was part of the study’s design, we cannot exclude that some of LSD’s effects may have influenced changes in the other conditions. Furthermore, it is unknown whether d-amphetamine and MDMA also induce mid/long-term changes in connectivity measures, which could, in turn, also influence the other conditions.

## Conclusions

By shedding light on the impact of LSD, d-amphetamine, and MDMA on large-scale connectivity patterns, we contribute to a more comprehensive understanding of the specific neural mechanisms underpinning psychedelics’ modes of action, potentially opening new avenues for the treatment of psychiatric disorders.

## Supplementary information

Supplemental Material

## Data Availability

This published article and its supplementary information files provide all data produced or examined during this research. Further data supporting the findings of this study can be obtained from the corresponding author upon reasonable request.

## Code availability

The preprocessing for this study was conducted using C-PAC. Additional code for computing network integration and segregation is provided in the supplement, see supplementary code.
