# Supplementary material for: Large-scale brain connectivity changes following the administration of lysergic acid diethylamide, d-amphetamine, and 3,4-methylenedioxyamphetamine
Source: Mol Psychiatry. 2024 Sep 11;30(4):1297–307. doi: 10.1038/s41380-024-02734-y (PMC11919773; doi:10.1038/s41380-024-02734-y)
Supplement: Supplementary file 1 — Supplemental Material [file 41380_2024_2734_MOESM1_ESM.docx]

**Supplementary Material for**

*“Large-scale brain connectivity changes following the administration of lysergic acid diethylamide, d-amphetamine, and 3,4-methylenedioxyamphetamine”*

By Avram et al.

[Supplementary methods 3](#_Toc175226470)

[Participants 3](#_Toc175226471)

[Study procedures 3](#_Toc175226472)

[Imaging parameters 4](#_Toc175226473)

[Supplementary results 5](#_Toc175226474)

[Seed-based correlation analysis 5](#_Toc175226475)

[Control for global signal regression 6](#_Toc175226476)

[Association between connectivity measures and 5D-ASC 8](#_Toc175226477)

[Supplementary discussion 10](#_Toc175226478)

[Global connectivity in psychedelic neuroimaging 10](#_Toc175226479)

[Control for global signal regression (GSR) 11](#_Toc175226480)

[Supplementary tables 14](#_Toc175226481)

[Table S1. Network integrity values for each condition. 14](#_Toc175226482)

[Table S2. One-way repeated-measures ANOVA for network segregation across the four conditions: LSD, d-amphetamine, MDMD, and placebo 15](#_Toc175226483)

[Table S3: Substance-specific changes in network segregation 16](#_Toc175226484)

[Table S4: Associations between substance-induced connectivity changes and changes in physiological parameters and head motion 18](#_Toc175226485)

[Table S5: Associations between substance-induced connectivity changes and subjective effects (11D-ASC) 20](#_Toc175226486)

[Table S6: Associations between substance-induced connectivity changes and subjective effects (5D-ASC) 23](#_Toc175226487)

[Table S7: Changes in global connectivity 25](#_Toc175226488)

[Supplementary figures 26](#_Toc175226489)

[Figure S1: Substance-induced changes in network integrity with GSR 26](#_Toc175226490)

[Figure S2: Substance-induced changes in network segregation with GSR 27](#_Toc175226491)

[Figure S3: Substance-specific changes in seed-based iFC 28](#_Toc175226492)

[Figure S4: Substance-induced changes in seed-based iFC with GSR 29](#_Toc175226493)

[Figure S5: Substance-specific changes in seed-based iFC with GSR 30](#_Toc175226494)

[Figure S6: Global functional connectivity with GSR 31](#_Toc175226495)

[Figure S7: The effects of GSR on within-condition differences in network segregation 32](#_Toc175226496)

[Supplementary Code 33](#_Toc175226497)

[Computing network integration (bash script) 33](#_Toc175226498)

[Computing network segregation (R script) 34](#_Toc175226499)

[References 35](#_Toc175226500)

# **Supplementary methods**

## **Participants**

Twenty-eight healthy volunteers (14 women, mean age 28 ± 4 years) were recruited for the study. All participants had no personal or first-degree relative history of major psychiatric disorders, as assessed through the Semi-structured Clinical Interview for DSM^1^. Exclusion criteria included pregnancy (verified via urine tests before each session), use of medications that could interact with the study substances (such as antipsychotics, antidepressants, and sedatives), presence of acute or chronic physical illnesses (based on a medical assessment), heavy smoking (more than 10 cigarettes per day), a lifetime history of illicit drug use exceeding ten instances (excluding cannabis), and illicit drug use during the study (verified through urine drug screenings). Participants were also instructed to limit alcohol consumption between sessions (less than 10 units per week, with no more than one unit on the day before a session) and to avoid xanthine-containing beverages after midnight before experimental sessions. Some participants had previous experiences with illicit substances, including those investigated in this study (refer to Holze et al.^2^ for details), but they were required to abstain from illicit drugs for at least two months prior to the study.

## **Study procedures**

Before inclusion in the study, participants underwent thorough medical and psychiatric screenings to confirm their health status. Once included, participants engaged in four 12-hour experimental sessions and one final study visit. An investigator accompanied each participant during the experimental sessions, providing interaction as needed (details in Holze et al.^2^).

Each subject received four treatments, administered orally (for detailed procedures, see Holze et al.^2^). Both subjects and study personnel were blinded to treatment order until the end-of-study visit. Identical vials and capsules were used for both the active substances and placebos. After each session and at the end of the study, participants were asked to guess their treatment assignments. An independent good manufacturing practice (GMP) facility (Apotheke Dr. Hysek, Biel, Switzerland) performed the randomization. The treatment order was random and counterbalanced. A treatment order was assigned to each participant number (code list) and kept by the GPM facility. Only the GMP facility and the study’s PIs had access to the code (i.e., in sealed envelopes).

Substances were administered at specific times to align with the peak effects during the fMRI scans: LSD and d-amphetamine or placebo at 9:00 a.m., and MDMA or placebo at 9:30 a.m. FMRI data collection occurred between 11:00 and 12:00 a.m.

## **Imaging parameters**

T1-weighted MRI images were obtained using the following specifications: repetition time (TR) of 2000 ms, echo time (TE) of 3.37 ms, flip angle (FA) of 8°, 176 slices, field of view (FoV) of 256 mm, matrix size of 256 × 256, and voxel size of 1.0 × 1.0 × 1.0 mm³.

Before the rs-fMRI sequence, participants were instructed to close their eyes while remaining awake. The acquisition involved three hundred volumes utilizing an interleaved T2*-weighted echo-planar imaging sequence, comprising 35 axial slices with a thickness of 3.5 mm and an intra-slice gap of 0.5 mm. The field of view was 224 × 224 mm, matrix size 64 × 64 (3.5 × 3.5 × 3.5 mm³ resolution), flip angle (FA) of 82°, repetition time (TR) of 1800 ms, and echo time (TE) of 28 ms.

# **Supplementary results**

## **Seed-based correlation analysis**

Compared to placebo, LSD decreased iFC within the VIS and increased iFC between the VIS and several other regions in the brain covering several transmodal networks (Figure 3). Similarly, the amphetamines also reduced within VIS iFC but unlike LSD, decreased the iFC between the VIS and areas covering the ASM. Similar to LSD, connectivity increases were found between the VIS and several transmodal networks. Regarding direct comparisons, we found that LSD induced stronger iFC between VIS and sensorimotor areas covering ASM than both amphetamines (Figure S3).

For SAL, we observed decreased within-network iFC for all substances compared to placebo, however, while the amphetamines decreased iFC in the anterior cingulum, LSD’s effects were restricted to the bilateral insula. LSD increased iFC between SAL and VIS, ASM, and DMN compared to placebo. Both amphetamines, when compared to placebo, increased iFC between SAL and VIS and DAN, respectively. In addition, when comparing the effects of the active substances, we observed that LSD exhibited increased iFC between SAL and several networks (e.g., VIS, ASM, and DMN) when compared to both amphetamines.

For FPN, all substances reduced iFC within the network, albeit with different patterns. Compared to placebo, LSD increased FPN iFC with VIS, ASM, and DMN regions. Compared to placebo, d-amphetamine increased FPN iFC with VIS, ASM, and DAN regions, but reduced iFC with some DMN regions. Similarly, MDMA increased FPN iFC with VIS and ASM regions, but reduced iFC with the thalamus, compared to placebo. When comparing the effects of the active substances, we observed that LSD induced widespread increases in FPN iFC with the majority of other networks, but reduced iFC within the FPN (e.g., angular gyrus) compared to the amphetamines.

For DMN, all substances induced decreased iFC within the network itself. For LSD this reduction was mainly observed in the posterior cingulum/precuneus. While LSD increased DMN iFC with several networks (both unimodal and transmodal), the amphetamines increased DMN iFC with unimodal networks (i.e., VIS and, for d-amphetamine, also ASM). Compared to the amphetamines, LSD increased DMN iFC with several other regions including areas belonging to ASM, DAN, and FPN.

For DAN, we observed higher iFC in ASM regions when comparing LSD to placebo. For d-amphetamine, DAN exhibited higher iFC with the FPN, in a small cluster, but lower iFC with the DMN, specifically in the left dorsal prefrontal cortex, compared to placebo. Notably, no significant iFC differences were found with MDMA compared to placebo. Regarding direct comparisons between the active substances, LSD exhibited stronger iFC between DAN and DMN, as well as within DAN itself, compared to both amphetamines. d-Amphetamine also exhibited higher iFC between DAN and the FPN compared to LSD, in a small cluster. Finally, MDMA showed higher iFC between DAN and DMN when compared to d-amphetamine.

Finally, regarding ASM, LSD induced lower iFC with the VIS, compared to placebo. ASM iFC was increased to DAN and FPN. Unlike LSD, both amphetamines reduced within ASM iFC extensively, compared to placebo. To a lesser extent, ASM iFC to VIS was also reduced by the amphetamines, compared to placebo. Additionally, the amphetamines increased ASM iFC with regions from the DMN and FPN. Furthermore, when comparing the effects of the active substances, we observed that LSD induced higher iFC within ASM and between ASM and VIS and DAN, respectively compared to both amphetamines. Finally, MDMA elicited higher iFC within ASM compared to d-amphetamine.

## **Control for global signal regression**

Considering the ongoing debate regarding the use of global signal regression (GSR) in psychedelic neuroimaging^3^, we tested the effects of this denoising procedure on all analyses.

1. Network Integrity

Including GSR in the analysis had noticeable effects on network integrity (Figure S1). Specifically, compared to placebo, we found decreased SAL and ASM integrity for LSD and decreased DMN integrity for the amphetamines. Furthermore, LSD induced lower integrity in the SAL and FPN than the two amphetamines, which was not observed in the analysis without GSR. Overall GSR induced greater reductions in network integrity for all substances with stronger effects observed for LSD.

1. Network Segregation

Including GSR as a preprocessing step significantly modified the results of the segregation analysis (Figure S2). As before, the correlation of the network pair ASM and DMN was not significantly different across conditions (F_3,96_=10.87, P_FDR_=0.80). However, the LSD pattern of connectivity changed with several network pairs now showing increased segregation (ASM and VIS, FPN and DAN, FPN and SAL)*,* which was not observed in the analysis without GSR. Additionally, several other network pairs no longer showed significantly decreased segregation (SAL and DAN, FPN both with ASM and DMN, DMN and DAN, DAN and VIS). The effects of the amphetamines were also influenced by GSR. While the substance-induced decreases in segregation remained unchanged, the segregation between SAL and DMN and SAL and FPN was increased.

Regarding direct comparisons between the active substances, the inclusion of GSR led to increased segregation for LSD compared to the amphetamines between the SAL and ASM and compared to amphetamine alone also between the SAL and DAN. Additional increases in segregation were observed for the FPN and ASM pair for LSD vs. MDMA. Additionally, previously significant decreases in segregation were no longer observed for the network pair SAL and FPN for LSD compared to both amphetamines and the pair SAL and DMN for LSD vs. MDMA only. Fewer changes were observed in the direct contrast between d-amphetamine and MDMA when using GSR. Specifically, while the decreased segregation between VIS and SAL was no longer significant for d-amphetamine vs. MDMA, we observed a significant decrease in segregation between the VIS and ASM.

1. Seed-based RSN iFC

Including GSR as a preprocessing step significantly modified the results of the seed-based iFC analysis (Figure S4 and S5). The results were mostly consistent with those from the analysis not including GSR, showing that when comparing all active substances to placebo most RSNs showed increased iFC with regions belonging to other RSNs, particularly between unimodal and transmodal networks, and decreased iFC within the seed network. However, GSR inclusion led to overall weaker effects across substances, more so for LSD than for d-amphetamine and MDMA. For instance, the connectivity of the DMN to the rest of the brain was no longer significant for LSD compared to placebo but increased for d-amphetamine and MDMA. The latter two also showed more extensive iFC for SAL in the analysis with GSR.

1. Degree centrality

Including GSR as a preprocessing step slightly modified the results of the global connectivity analysis (Figure S6). In contrast to the analysis without GSR, the d-amphetamine vs. MDMA contrast turned significant. The other contrasts, active substance vs. placebo and vice versa, remained largely consistent.

## **Association between connectivity measures and 5D-ASC**

Considering that some clinical studies have examined associations between the five-dimension measures (5D-ASC) and clinical outcomes^4-6^, we additionally computed correlations between substance-induced connectivity changes and the items of the 5D-ASC instead of 11D-ASC. We used the same partial correlation analysis approach as before to explore the relationships between the investigated connectivity metrics and subjective effects while controlling for physiological parameters and head motion. The associations are detailed in Table S6.

- LSD: Changes in network integrity and decreased DC correlated with subjective effects. DMN integrity was associated with the most subjective effects.
- d-Amphetamine: SAL integrity was associated with most factors, similar to the correlations with the 11 factors. Additionally, ASM iFC correlated with the factor of auditory alterations.
- MDMA: Only DAN integrity correlated with subjective effects, which differed from the 11 factors’ findings.

# **Supplementary discussion**

## **Global connectivity in psychedelic neuroimaging**

Previous studies investigated the effects of LSD^7-9^, psilocybin^8, 10^, and DMT^11^ on global connectivity. While all studies, including our own, assess a form of global connectivity, there are important methodological differences that may help explain the discrepant results across studies (see Table below). In the following, we primarily focus on LSD studies.

First, there are differences in study design. LSD was administered intravenously in the Tagliazucchi et al. study^8^, which could be linked to slightly different pharmacodynamics. Furthermore, while Tagliazucchi and colleagues^8^ had two conditions (i.e., LSD vs. placebo), akin to Müller and colleagues^9^, Preller et al^7^ had a design with three conditions (placebo, LSD, and LSD + ketanserin), and the current study had four (placebo, LSD, d-amphetamine, and MDMA). Due to the cross-over design, we cannot exclude carry-over effects; it is therefore possible that the d-amphetamine or MDMA conditions affected the LSD vs. placebo contrast.

Second, the studies used different methods and metrics to investigate global connectivity. Tagliazucchi and colleagues^8^ computed functional connectivity density (FCD) as the average correlation between time series of 401 ROIs. They observed increased FCD for frontal, parietal, inferior temporal cortices, the basal ganglia, and the thalamus in the LSD>placebo contrast. The cortical areas showing increased connectivity covered areas of the FPN, DMN, and SAL (see Figure 1, Panels C and D in Tagliazucchi et al^8^). No areas with decreased FCD were reported. Preller and colleagues^7^ investigated global connectivity by computing the global brain connectivity (GBC) metric with in-house scripts. Like FCD, GBC computes connectivity from a given region (or voxel) to all other regions/voxels simultaneously by computing average connectivity strength. Unlike Tagliazucchi et al^8^, Preller and colleagues^7^ performed the analyses both without and with an additional preprocessing/denoising step, namely global signal regression (GSR). Without GSR, in the LSD vs. placebo contrast, Preller et al^7^ found increased GBC in the cerebellum, basal ganglia, thalamus, and in small clusters covering parts of the posterior cingulum/precuneus; decreased GBC was observed in visual, sensorimotor, and insular cortices (Figure 2, panel C in Preller et al^7^). However, when including GSR, Preller and colleagues found the opposite pattern, namely increased GBC in visual and sensorimotor areas, and decreased GBC in association areas (i.e., prefrontal, parietal, etc.), the basal ganglia, and the thalamus (Figure 2, panel C in Preller et al^7^). Finally, Müller and colleagues^9^ computed global correlation (GCOR) with the CONN toolbox. Similar to the other measures, GCOR is a data-driven, whole-brain measure reflecting the averaged correlation coefficients of each voxel to each other voxel. Increased GCOR was found in the basal ganglia and the thalamus (Figure 3 in Müller and colleagues^9^). No decreases were reported.

Despite using somewhat different approaches, global connectivity was computed across studies (including the present one) by extracting the time series of one voxel or region and correlating it with the time series of all other voxels/regions in the brain, followed by averaging the correlation coefficients. An important difference in our approach with DC is that correlations are thresholded before averaging, which help to better control for noise and spurious correlations. Specifically, we only kept significant correlations (P<0.001) in our analyses. Nevertheless, despite this methodological difference, the current findings are akin to those of Müller and colleagues^9^, where no thresholding was applied. Notably, across studies, the most consistent finding was increased global connectivity in the basal ganglia and thalamus, which is also consistent with studies investigating other serotonergic psychedelics such as psilocybin^8, 10^ and DMT – as long as GSR was not employed^11^.

## **Control for global signal regression (GSR)**

Global signal regression (GSR) is a controversial preprocessing procedure that removes the time series of signal intensity averaged across all brain voxels through linear regression^12^. The global signal is believed to include several non-neuronal artifacts such as physiological, movement, or scanner-related parameters^13^. However, GSR can induce spurious anticorrelations in the data^12^ and may also remove meaningful neural information^14^.

Our results demonstrate that including GSR in preprocessing leads to considerable changes across all analyses. Specifically, GSR resulted in stronger reductions in network integrity, more extensive between-network segregation (i.e., decreased connectivity), and weaker seed-based RSN iFC compared to placebo. Notably, these effects were more pronounced for LSD than for d-amphetamine or MDMA, as observed in the direct contrasts between the active substances.

The active substances differentially affect physiological parameters linked to the global signal. For instance, d-amphetamine and MDMA increase systolic and diastolic blood pressure more than LSD, while LSD increases heart rate more than the amphetamines; additionally, the substances have distinct psychological effects^2^, likely tied to differential neural processing. It is plausible that these differences in physiological and neural activity alter the content of the global signal, thus differentially impacting the effects of GSR.

To explore this, we performed within-condition comparisons of the correlation coefficients between RSN pairs for each substance and placebo separately. Without exception, all correlations were weaker following GSR (Figure S7). Consistent with our previous findings, the difference in correlation coefficients was largest for LSD, followed by d-amphetamine, MDMA, and lastly placebo. Importantly, controlling for heart rate, systolic and diastolic blood pressure, temperature, and mean framewise displacement did not alter these results, suggesting that the differential impact of GSR is not solely due to these physiological differences and head motion.

Moreover, GSR affected both the conditions and RSNs differently. While d-amphetamine, MDMA, and placebo exhibited the highest changes in similar network pairs with varying intensities (e.g., VIS and FPN, DMN and DAN), in the LSD condition other network pairs were more affected (e.g., DMN and ASM, DMN and SAL). Our findings indicate the need for careful consideration when applying GSR, as it can significantly alter the outcomes and may not uniformly affect all conditions under study.

Based on our findings, we caution against the routine use of GSR in psychedelic neuroimaging studies. The significant and differential effects observed suggest that its application could obscure true neural signals and potentially introduce biases, particularly in studies involving substances with distinct physiological and psychological effects.

# **Supplementary tables**

## **Table S1.** Network integrity values for each condition.

| **Without GSR** | | | | |
| --- | --- | --- | --- | --- |
| **RSN** | **Placebo** | **LSD** | **d-Amph.** | **MDMA** |
| **ASM** * | 2.93 (0.5) | 2.87 (0.58) | 1.95 (0.48) | 2.21 (0.58) |
| **DAN** | 3.09 (0.48) | 3.18 (0.68) | 3.05 (0.54) | 3.08 (0.56) |
| **DMN** * | 3.32 (0.57) | 2.92 (0.62) | 3.09 (0.53) | 3.04 (0.6) |
| **FPN** * | 3.1 (0.51) | 2.59 (0.55) | 2.77 (0.31) | 2.7 (0.44) |
| **SAL** | 2.86 (0.34) | 2.64 (0.54) | 2.54 (0.49) | 2.56 (0.45) |
| **VIS** * | 5.15 (1.09) | 3.46 (0.59) | 3 (0.75) | 3.63 (1.2) |
| **With GSR** | | | | |
| **RSN** | **Placebo** | **LSD** | **d-Amph.** | **MDMA** |
| **ASM** * | 2.65 (045) | 2.24 (0.47) | 1.70 (0.34) | 1.88 (0.48) |
| **DAN** | 2.76 (0.57) | 2.42 (0.60) | 2.70 (0.53) | 2.52 (0.54) |
| **DMN** * | 2.64 (0.39) | 2.14 (0.52) | 2.34 (0.39) | 2.35 (0.58) |
| **FPN** * | 2.65 (0.49) | 1.77 (0.41) | 1.99 (0.29) | 2.03 (0.42) |
| **SAL *** | 2.60 (0.42) | 1.91 (0.49) | 2.18 (0.31) | 2.24 (0.48) |
| **VIS** * | 4.63 (1.12) | 2.55 (0.64) | 2.23 (0.66) | 2.96 (1.21) |

Integrity values are shown as mean and standard deviation. Repeated-measures ANOVAs were computed on dual regression-derived integrity values and corrected for multiple comparisons (P_FDR_<.05). * - depicts significant differences between conditions. Abbreviations: VIS – visual network, SAL – salience network, FPN – frontoparietal network, DMN – default mode network, ASM – auditory-sensorimotor network.

## **Table S2.** One-way repeated-measures ANOVA for network segregation across the four conditions: LSD, d-amphetamine, MDMD, and placebo

| **RSN1** | **RSN2** | **F-value** | **p** | **FDR** |
| --- | --- | --- | --- | --- |
| **ASM** | **DAN** | 16.75 | 2.35E-08 | 8.81E-08 |
|  | **DMN** | 2.54 | 0.063 | 0.063 |
|  | **FPN** | 8.12 | 9.95E-05 | 1.87E-04 |
|  | **SAL** | 20.93 | 7.45E-10 | 3.73E-09 |
|  | **VIS** | 3.94 | 0.012 | 0.0129 |
| **DAN** | **DMN** | 7.85 | 1.32E-04 | 2.20E-04 |
|  | **FPN** | 6.81 | 4.18E-04 | 5.70E-04 |
|  | **SAL** | 4.73 | 0.005 | 6.25E-03 |
|  | **VIS** | 10.67 | 6.92E-06 | 1.73E-05 |
| **DMN** | **FPN** | 13.36 | 5.05E-07 | 1.52E-06 |
|  | **SAL** | 7.12 | 2.98E-04 | 4.47E-04 |
|  | **VIS** | 9.71 | 1.84E-05 | 3.94E-05 |
| **FPN** | **SAL** | 3.93 | 0.012 | 0.0129 |
|  | **VIS** | 25.14 | 3.09E-11 | 2.32E-10 |
| **SAL** | **VIS** | 25.77 | 1.97E-11 | 2.32E-10 |

## **Table S3:** Substance-specific changes in network segregation

|  | **LSD vs. d-Amphetamine** | **LSD vs. MDMA** | **d-Amphetamine vs. MDMA** |
| --- | --- | --- | --- |
| **VIS~ASM** |  |  |  |
| **VIS~DAN** | **↑** |  | **↓** |
| **VIS~DMN** | **↓** |  |  |
| **VIS~FPN** |  |  |  |
| **VIS~SAL** |  | **↑** | **↑** |
| **SAL~ASM** |  |  |  |
| **SAL~DAN** |  |  |  |
| **SAL~DMN** | **↑** | **↑** |  |
| **SAL~FPN** | **↑** | **↑** |  |
| **SAL~VIS** |  | **↑** | **↑** |
| **FPN~ASM** |  |  |  |
| **FPN~DAN** | **↓** |  | **↑** |
| **FPN~DMN** | **↑** | **↑** | **↓** |
| **FPN~SAL** | **↑** | **↑** |  |
| **FPN~VIS** |  |  |  |
| **DMN~ASM** |  |  |  |
| **DMN~DAN** | **↑** |  | **↓** |
| **DMN~FPN** | **↑** | **↑** | **↓** |
| **DMN~SAL** | **↑** | **↑** |  |
| **DMN~VIS** | **↓** |  |  |
| **DAN~ASM** | **↑** | **↑** |  |
| **DAN~DMN** | **↑** |  | **↓** |
| **DAN~FPN** | **↓** |  | **↑** |
| **DAN~SAL** |  |  |  |
| **DAN~VIS** | **↑** |  | **↓** |
| **ASM~DAN** | **↑** | **↑** |  |
| **ASM~DMN** |  |  |  |
| **ASM~FPN** |  |  |  |
| **ASM~SAL** |  |  |  |
| **ASM~VIS** |  |  |  |

Depicted are increases (red arrows) and decreases (blue arrows) between pairs of RSNs iFC. Only significant results are shown (P_FDR_<.05).

## **Table S4:** Associations between substance-induced connectivity changes and changes in physiological parameters and head motion

| **LSD-induced changes** | | | | | |
| --- | --- | --- | --- | --- | --- |
|  | **∆HR** | **∆SBP** | **∆DPB** | **∆Temp** | **∆FD** |
| **VIS integrity** | r=-.26 p=.20 | r=.12  p=.56 | r=.40  p=.04* | r=.05  p=.81 | r=.18  p=.38 |
| **VIS iFC** | r=-.27 p=.18 | r=.14  p=.49 | r=.36  p=.06 | r=-.06 p=.76 | r=-.00 p=.97 |
| **SAL integrity** | r=-.17 p=.39 | r=.15  p=.45 | r=.14  p=.50 | r=-.07 p=.71 | r=.15  p=.45 |
| **SAL iFC** | r=-.43 p=.03* | r=.14  p=.47 | r=.34  p=.09 | r=-.12 p=.54 | r=-.02 p=.90 |
| **FPN integrity** | r=.09  p=.66 | r=.43  p=.03* | r=.16  p=.42 | r=.02  p=.89 | r=.13  p=.53 |
| **FPN iFC** | r=-.34 p=.09 | r=.12  p=.55 | r=.38  p=.05 | r=.03  p=.87 | r=.02  p=.90 |
| **DMN integrity** | r=.04  p=.81 | r=.23  p=.26 | r=.04  p=.84 | r=.03  p=.86 | r=.31  p=.12 |
| **DMN iFC** | r=-.29 p=.15 | r=.22  p=.29 | r=.32  p=.11 | r=-.09 p=.64 | r=-.00 p=.97 |
| **DAN integrity** | r=-.02 p=.88 | r=.05  p=.78 | r=.17  p=.40 | r=.02  p=.91 | r=-.02 p=.88 |
| **DAN iFC** | r=-.42 p=.03* | r=.13  p=.52 | r=.13  p=.53 | r=-.27 p=.17 | r=-.13  p=.50 |
| **ASM integrity** | r=-.34 p=.09 | r=-.08 p=.68 | r=-.16 p=.42 | r=-.16 p=.43 | r=-.01 p=.94 |
| **ASM iFC** | r=-.18 p=.37 | r=.13  p=.53 | r=.39  p=.04* | r=.02  p=.91 | r=.02  p=.92 |
| **LSD>PCB DC** | r=.08  p=.69 | r=.03  p=.85 | r=-.05  p=.79 | r=.00  p=.98 | r=-.24 p=.23 |
| **PCB>LSD DC** | r=-.37 p=.06 | r=-.25 p=.22 | r=-.05  p=.79 | r=-.67  p<.001** | r=.34  p=.09 |
| **d-Amphetamine-induced changes** | | | | | |
|  | **∆HR** | **∆SBP** | **∆DPB** | **∆Temp** | **∆FD** |
| **VIS integrity** | r=-.00 p=.99 | r=-.39 p=.05 | r=-.35 p=.08 | r=-.33 p=.10 | r=.20  p=.31 |
| **VIS iFC** | r=-.30 p=.14 | r=-.17 p=.39 | r=-.20 p=.32 | r=.21  p=.31 | r=.40  p=.04* |
| **SAL integrity** | r=-.09 p=.65 | r=-.25 p=.21 | r=-.08 p=.67 | r=-.10 p=.62 | r=.49  p=.01* |
| **SAL iFC** | r=-.13 p=.53 | r=-.12 p=.55 | r=.02  p=.91 | r=.04  p=.83 | r=.24  p=.23 |
| **FPN integrity** | r=-.12 p=.54 | r=-.15 p=.47 | r=-.05  p=.78 | r=-.03 p=.88 | r=.36  p=.07 |
| **FPN iFC** | r=-.31 p=.14 | r=-.13 p=.52 | r=-.32 p=.11 | r=.01  p=.95 | r=.43  p=.03* |
| **DMN integrity** | r=-.29 p=.15 | r=-.34 p=.08 | r=-.38 p=.06 | r=-.00  p=.98 | r=.43  p=.03* |
| **DMN iFC** | r=-.21 p=.30 | r=-.30  p=.14 | r=-.16 p=.43 | r=.16  p=.43 | r=.27  p=.19 |
| **DAN integrity** | r=-.22 p=.27 | r=-.11  p=.58 | r=-.19 p=.36 | r=-.18 p=.37 | r=.18  p=.37 |
| **DAN iFC** | r=-.09 p=.66 | r=-.31 p=.14 | r=-.26 p=.19 | r=-.21 p=.30 | r=.18  p=.38 |
| **ASM integrity** | r=-.08 p=.68 | r=-.14 p=.50 | r=-.22 p=.28 | r=-.09 p=.65 | r=-.48 p=.01* |
| **ASM iFC** | r=-.08 p=.69 | r=-.30 p=.14 | r=-.29 p=.14 | r=-.10 p=.60 | r=.35  p=.08 |
| **AMP>PCB DC** | r=.24  p=.24 | r=.06  p=.77 | r=.21  p=.29 | r=.26  p=.19 | r=-.25 p=.22 |
| **PCB>AMP DC** | r=-.21  p=.29 | r=-.31  p=.13 | r=-.03 p=.88 | r=-.42 p=.03* | r=.14  p=.48 |
| **MDMA-induced changes** | | | | | |
|  | **∆HR** | **∆SBP** | **∆DPB** | **∆Temp** | **∆FD** |
| **VIS integrity** | r=-.02  p=.91 | r=.12  p=.55 | r=.12  p=.54 | r=-.10  p=.62 | r=.03  p=.88 |
| **VIS iFC** | r=-.29  p=.14 | r=-.08  p=.70 | r=-.04  p=.83 | r=-.09  p=.65 | r=.17  p=.40 |
| **SAL integrity** | r=-.22  p=.27 | r=.17  p=.40 | r=.19  p=.36 | r=.05  p=.80 | r=.42  p=.03* |
| **SAL iFC** | r=-.13  p=.52 | r=.21  p=.30 | r=.18  p=.38 | r=-.01  p=.95 | r=-.05  p=.79 |
| **FPN integrity** | r=-.04  p=.82 | r=.25  p=.21 | r=.26  p=.19 | r=-.08  p=.68 | r=.42  p=.03* |
| **FPN iFC** | r=-.26  p=.20 | r=-.04  p=.84 | r=.06  p=.75 | r=.05  p=.79 | r=.15  p=.47 |
| **DMN integrity** | r=-.35  p=.08 | r=-.06  p=.74 | r=-.12  p=.54 | r=-.09  p=.66 | r=.36  p=.07 |
| **DMN iFC** | r=-.21  p=.30 | r=-.10  p=.60 | r=-.01  p=.96 | r=.13  p=.51 | r=.18  p=.36 |
| **DAN integrity** | r=-.30  p=.14 | r=.00  p=.96 | r=-.00  p=.96 | r=-.28  p=.17 | r=.29  p=.15 |
| **DAN iFC** | - | - | - | - | - |
| **ASM integrity** | r=-.28  p=.17 | r=.27  p=.17 | r=.30  p=.14 | r=.13  p=.53 | r=.24  p=.23 |
| **ASM iFC** | r=-.08  p=.67 | r=-.08  p=.69 | r=.03  p=.88 | r=-.11  p=.60 | r=.22  p=.28 |
| **MDMA>PCB DC** | r=.06  p=.76 | r=-.31  p=.12 | r=-.29  p=.15 | r=-.06  p=.76 | r=.08  p=.69 |
| **PCB>MDMA DC** | r=-.26  p=.20 | r=-.55  p=.004* | r=-.48  p=.01 | r=-.45  p=.02* | r=.17  p=.40 |

## **Table S5:** Associations between substance-induced connectivity changes and subjective effects (11D-ASC)

| **Associations between LSD-induced changes and 11D-ASC** | | | | | | | | | | | |
| --- | --- | --- | --- | --- | --- | --- | --- | --- | --- | --- | --- |
|  | **EoU** | **SE** | **BS** | **I** | **D** | **ICaC** | **A** | **CI** | **EI** | **AVS** | **CmoP** |
| **VIS integrity** | r=.04  p=.86 | r=.23  p=.31 | r=.16  p=.49 | r=-.07  p=.75 | r=-.08  p=.72 | r=-.19  p=.40 | r=-.00  p=.99 | r=-.31  p=.17 | r=-.14  p=.55 | r=.01  p=.94 | r=.07  p=.74 |
| **VIS iFC** | r=-.02  p=.92 | r=.27  p=.23 | r=.11  p=.63 | r=.20  p=.37 | r=.15  p=.51 | r=.14  p=.53 | r=.21  p=.37 | r=.06  p=.79 | r=-.13  p=.57 | r=.36  p=.11 | r=.18  p=.42 |
| **SAL integrity** | r=-.06  p=.80 | r=.25  p=.28 | r=.16  p=.48 | r=-.04  p=.84 | r=-.25  p=.28 | r=-.26  p=.25 | r=.29  p=.21 | r=-.29  p=.21 | r=-.18  p=.42 | r=.27  p=.24 | r=.14  p=.54 |
| **SAL iFC** | r=-.10  p=.65 | r=.05  p=.83 | r=.02  p=.92 | r=.05  p=.82 | r=.12  p=.61 | r=.16  p=.48 | r=.29  p=.21 | r=-.01  p=.93 | r=-.20  p=.38 | r=.30  p=.18 | r=.32  p=.17 |
| **FPN integrity** | r=-.43  p=.05 | r=.17  p=.46 | r=-.29  p=.20 | r=-.25  p=.28 | r=-.32  p=.16 | r=-.09  p=.70 | r=.34  p=.13 | r=-.48  p=.03 | r=-.20  p=.38 | r=-.08  p=.71 | r=-.06  p=.80 |
| **FPN iFC** | r=.15  p=.51 | r=.30  p=.18 | r=.32  p=.16 | r=.20  p=.38 | r=.38  p=.09 | r=.24  p=.30 | r=.01  p=.94 | r=.23  p=.33 | r=-.10  p=.65 | r=.34  p=.13 | r=.29  p=.33 |
| **DMN integrity** | r=-.48  p=.02 | r=.14  p=.55 | r=-.25  p=.27 | r=-.44  p=.05 | r=-.40  p=.08 | r=-.21  p=.35 | r=.05  p=.80 | r=-.52  p=.01 | r=-.27  p=.24 | r=-.36  p=.11 | r=-.19  p=.40 |
| **DMN iFC** | r=.11  p=.64 | r=.19  p=.41 | r=.20  p=.39 | r=.23  p=.32 | r=.39  p=.04 | r=.31  p=.17 | r=.26  p=.26 | r=.27  p=.24 | r=-.01  p=.93 | r=.33  p=.14 | r=.13  p=.54 |
| **DAN integrity** | r=-.38  p=.09 | r=.09  p=.68 | r=-.01  p=.94 | r=-.31  p=.18 | r=-.36  p=.11 | r=-.06  p=.80 | r=.28  p=.23 | r=-.26  p=.25 | r=-.14  p=.52 | r=-.21  p=.35 | r=-.23  p=.32 |
| **DAN iFC** | r=-.22  p=.34 | r=.04  p=.84 | r=-.04  p=.84 | r=-.24  p=.30 | r=-.20  p=.38 | r=-.15  p=.52 | r=.06  p=.79 | r=-28.  p=.22 | r=-.39  p=.08 | r=-.12  p=.61 | r=-.26  p=.26 |
| **ASM integrity** | r=-.30  p=.18 | r=-.05  p=.80 | r=-.10  p=.67 | r=-.27  p=.25 | r=-.55  p=.01 | r=-37  p=.10 | r=.05  p=.83 | r=-.42  p=.05 | r=-.39  p=.08 | r=-.21  p=.35 | r=-.32  p=.15 |
| **ASM iFC** | r=-.05  p=.80 | r=.16  p=.49 | r=.16  p=.49 | r=-.06  p=.74 | r=.17  p=.46 | r=.27  p=.23 | r=.26  p=.26 | r=.09  p=.70 | r=-.08  p=.71 | r=.22  p=.35 | r=.27  p=.23 |
| **LSD>**  **PCB DC** | r=.21  p=.30 | r=-.17  p=.41 | r=.14  p=.50 | r=.07  p=.73 | r=-.07  p=.72 | r=-.22  p=.28 | r=-.04  p=.84 | r=-.01  p=.93 | r=-.10  p=.65 | r=.26  p=.19 | r=.38  p=.06 |
| **PCB>**  **LSD DC** | r=-.06  p=.75 | r=-.14  p=.49 | r=-.02  p=.89 | r=-.13  p=.52 | r=-.03  p=.87 | r=.23  p=.26 | r=-.19  p=.36 | r=-.02  p=.89 | r=.12  p=.55 | r=-.10  p=.61 | r=-.03  p=.87 |
| **Associations between d-amphetamine-induced changes and 11D-ASC** | | | | | | | | | | | |
|  | **EoU** | **SE** | **BS** | **I** | **D** | **ICaC** | **A** | **CI** | **EI** | **AVS** | **CmoP** |
| **VIS integrity** | r=-.09  p=.69 | r=-.17  p=.42 | r=-.02  p=.93 | r=-.14  p=.53 | r=-.13  p=.58 | r=.32  p=.16 | r=-.06  p=.77 | r=-.20  p=.39 | r=-.06  p=.77 | r=-.06  p=.77 | r=.08  p=.72 |
| **VIS iFC** | r=.16  p=.50 | r=-.14  p=.55 | r=-.13  p=.58 | r=-.36  p=.11 | r=-.37  p=.10 | r=.22  p=.35 | r=.18  p=.43 | r=-.05  p=.81 | r=.18  p=.43 | r=.18  p=.43 | r=-.24  p=.29 |
| **SAL integrity** | r=-.03  p=.88 | r=-.17  p=.47 | r=-.36  p=.11 | r=-.47  p=.03 | r=-.44  p=.04 | r=-.16  p=.49 | r=.21  p=.36 | r=-.15  p=.51 | r=.21  p=.36 | r=.21  p=.36 | r=-.54  p=.01 |
| **SAL iFC** | r=-.06  p=.79 | r=-.32  p=.16 | r=.02  p=.93 | r=-.18  p=.43 | r=-.27  p=.23 | r=.12  p=.59 | r=.04  p=.86 | r=.03  p=.89 | r=.04  p=.86 | r=.04  p=.86 | r=-.24  p=.29 |
| **FPN integrity** | r=-.05  p=.80 | r=-.05  p=.83 | r=-.33  p=.15 | r=-.34  p=.13 | r=-.44  p=.04 | r=-.00  p=.99 | r=.00  p=.99 | r=-.13  p=.58 | r=.00  p=.99 | r=.00  p=.99 | r=-.40  p=.07 |
| **FPN iFC** | r=.28  p=.23 | r=-.11  p=.62 | r=-.07  p=.74 | r=-.33  p=.15 | r=-.42  p=.06 | r=.40  p=.08 | r=.18  p=.43 | r=.05  p=.81 | r=.18  p=.43 | r=.18  p=.43 | r=-.08  p=.71 |
| **DMN integrity** | r=-.12  p=.61 | r=-.04  p=.86 | r=-.05  p=.81 | r=.16  p=.48 | r=.25  p=.27 | r=.00  p=.98 | r=-.04  p=.84 | r=.06  p=.77 | r=-.04  p=.84 | r=-.04  p=.84 | r=.14  p=.53 |
| **DMN iFC** | r=.13  p=.57 | r=-.12  p=.60 | r=-.09  p=.69 | r=-.36  p=.11 | r=-.34  p=.14 | r=.28  p=.22 | r=.14  p=.53 | r=-.20  p=.39 | r=.14  p=.53 | r=.14  p=.53 | r=-.08  p=.73 |
| **DAN integrity** | r=-.61  p=004 | r=-.25  p=.28 | r=-.34  p=.13 | r=-.23  p=.32 | r=-.10  p=.68 | r=-.25  p=.28 | r=-.36  p=.11 | r=-.35  p=.12 | r=-.36  p=.11 | r=-.36  p=.11 | r=-.36  p=.11 |
| **DAN iFC** | r=.14  p=.53 | r=-.01  p=.95 | r=-.29  p=.21 | r=-.29  p=.21 | r=-.16  p=.48 | r=-.20  p=.38 | r=.31  p=.17 | r=-.20  p=.39 | r=.31  p=.17 | r=.31  p=.17 | r=-32.  p=.15 |
| **ASM integrity** | r=-.05  p=.80 | r=-.27  p=.24 | r=-.32  p=.09 | r=-.50  p=.02 | r=-.34  p=.14 | r=-.12  p=.61 | r=.26  p=.25 | r=-.20  p=.39 | r=.26  p=.25 | r=.26  p=.25 | r=-.46  p=.03 |
| **ASM iFC** | r=.38  p=.09 | r=-.02  p=.92 | r=.08  p=.72 | r=-.30  p=.19 | r=-.43  p=.05 | r=.32  p=.16 | r=.43  p=.05 | r=.02  p=.91 | r=.43  p=.05 | r=.43  p=.05 | r=-.15  p=.51 |
| **Amph.>**  **PCB DC** | r=-.38  p=.06 | r=-.21  p=.29 | r=.02  p=.90 | r=-.03  p=.85 | r=-.03  p=.87 | r=-.06  p=.77 | r=-.25  p=.22 | r=-.09  p=.66 | r=-.25  p=.22 | r=-.25  p=.22 | r=-.11  p=.60 |
| **PCB>**  **Amph. DC** | r=-.20  p=.33 | r=-.08  p=.67 | r=-.38  p=.06 | r=-.16  p=.44 | r=-.07  p=.72 | r=.05  p=.79 | r=-.01  p=.95 | r=-.23  p=.26 | r=-.01  p=.95 | r=-.01  p=.95 | r=.28  p=.16 |
| **Associations between MDMA-induced changes and 11D-ASC** | | | | | | | | | | | |
|  | **EoU** | **SE** | **BS** | **I** | **D** | **ICaC** | **A** | **CI** | **EI** | **AVS** | **CmoP** |
| **VIS integrity** | r=.43  p=.05 | r=.37  p=.10 | r=.39  p=.08 | r=.15  p=.50 | r=.25  p=.28 | r=.08  p=.71 | r=-.22  p=.34 | r=.20  p=.39 | r=-.05  p=.82 | r=.08  p=.71 | r=.34  p=.13 |
| **VIS iFC** | r=-.20  p=.39 | r=-.12  p=.59 | r=-.27  p=.23 | r=-.00  p=.97 | r=-.12  p=.58 | r=-.30  p=.18 | r=.19  p=.42 | r=-.06  p=.79 | r=.26  p=.25 | r=.13  p=.57 | r=-.19  p=.41 |
| **SAL integrity** | r=-.02  p=.90 | r=-.01  p=.94 | r=.16  p=.47 | r=-.10  p=.67 | r=.30  p=.19 | r=-.08  p=.72 | r=-.31  p=.17 | r=-.07  p=.76 | r=.11  p=.62 | r=.16  p=.48 | r=-.05  p=.81 |
| **SAL iFC** | r=.13  p=.56 | r=.14  p=.53 | r=.30  p=.19 | r=.03  p=.89 | r=.24  p=.30 | r=-.10  p=.65 | r=-.02  p=.92 | r=.02  p=.92 | r=.20  p=.38 | r=.16  p=.50 | r=.08  p=.72 |
| **FPN integrity** | r=.13  p=.57 | r=.16  p=.49 | r=.24  p=.30 | r=.05  p=.82 | r=.16  p=.47 | r=.29  p=.20 | r=.02  p=.92 | r=.11  p=.64 | r=.06  p=.79 | r=.21  p=.36 | r=.15  p=.50 |
| **FPN iFC** | r=-.14  p=.54 | r=-.10  p=.65 | r=-.19  p=.40 | r=-.03  p=.86 | r=-.14  p=.54 | r=-.32  p=.16 | r=.12  p=.59 | r=-.09  p=.69 | r=.23  p=.32 | r=.09  p=.69 | r=-.19  p=.42 |
| **DMN integrity** | r=.12  p=.59 | r=.14  p=.54 | r=.23  p=.34 | r=.22  p=.33 | r=.18  p=.44 | r=.05  p=.80 | r=-.53  p=.01 | r=.16  p=.48 | r=-.22  p=.35 | r=-.10  p=.67 | r=.26  p=.25 |
| **DMN iFC** | r=.07  p=.76 | r=.16  p=.50 | r=-.21  p=.36 | r=.15  p=.52 | r=-.17  p=.47 | r=-.32  p=.16 | r=.29  p=.20 | r=.12  p=.60 | r=.21  p=.36 | r=.20  p=.39 | r=-.07  p=.75 |
| **DAN integrity** | r=.38  p=.14 | r=.26  p=.26 | r=.44  p=.05 | r=.22  p=.33 | r=.42  p=.06 | r=-.00  p=.99 | r=-.28  p=.22 | r=.21  p=.35 | r=-.01  p=.95 | r=.04  p=.83 | r=.27  p=.24 |
| **DAN iFC** | - | - | - | - | - | - | - | - | - | - | - |
| **ASM integrity** | r=.24  p=.30 | r=.27  p=.23 | r=.11  p=.62 | r=.06  p=.78 | r=.14  p=.53 | r=-.32  p=.15 | r=-.46  p=.04 | r=.08  p=.71 | r=.06  p=.77 | r=.10  p=.66 | r=-.01  p=.94 |
| **ASM iFC** | r=-.10  p=.66 | r=-15.  p=.51 | r=.08  p=.73 | r=-.03  p=.87 | r=.09  p=.68 | r=-.03  p=.88 | r=.16  p=.47 | r=-.07  p=.76 | r=.11  p=.63 | r=.07  p=.75 | r=.01  p=.96 |
| **MDMA>**  **PCB DC** | r=-.31  p=.18 | r=-25.  p=.27 | r=-.30  p=.19 | r=-.03  p=.88 | r=-.11  p=.63 | r=-.29  p=.20 | r=-.29  p=.21 | r=-.13  p=.58 | r=.19  p=.41 | r=.05  p=.83 | r=-.22  p=.34 |
| **PCB>**  **MDMA DC** | r=.00  p=.98 | r=.11  p=.63 | r=-.33  p=.15 | r=.13  p=.58 | r=-.04  p=.85 | r=-.30  p=.19 | r=.05  p=.82 | r=.12  p=.61 | r=.13  p=.57 | r=.31  p=.17 | r=.05  p=.81 |

Orange cells depict trend associations between connectivity measures and items of the 11D-ASC. Correlations were not corrected for multiple comparisons. Green cells depict items of the 11D-ASC which showed little to no variance (e.g., all values were 0). As MDMA-related DAN iFC did not differ from placebo, no connectivity changes were available for association with subjective measures in this case. While trend associations between subjective effects and connectivity changes were found for both d-amphetamine and MDMA, we note that these compounds did not significantly alter subjective effects, as measured by the 11D-ASC, compared to placebo.

## **Table S6:** Associations between substance-induced connectivity changes and subjective effects (5D-ASC)

| **Associations between LSD-induced changes and 5D-ASC** | | | | | |
| --- | --- | --- | --- | --- | --- |
|  | **OSE** | **AIA** | **VUS** | **AUD** | **VIG** |
| **VIS integrity** | r=.03  p=.88 | r=-.10  p=.65 | r=-.12  p=.59 | r=-.24  p=.29 | r=-.19  p=.40 |
| **VIS iFC** | r=.09  p=.68 | r=.19  p=.40 | r=.12  p=.58 | r=.09  p=.69 | r=-.05  p=.83 |
| **SAL integrity** | r=-.03  p=.88 | r=-.11  p=.62 | r=-.05  p=.81 | r=-.40  p=.07 | r=-.27  p=.23 |
| **SAL iFC** | r=.01  p=.95 | r=.32  p=.18 | r=.11  p=.62 | r=.15  p=.50 | r=-.06  p=.79 |
| **FPN integrity** | r=-.41  p=.06 | r=.09  p=.69 | r=-.28  p=.22 | r=-.38  p=.09 | r=-.22  p=.33 |
| **FPN iFC** | r=.32  p=.18 | r=.16  p=.49 | r=.19  p=.40 | r=.28  p=.22 | r=.03  p=.87 |
| **DMN integrity** | r=-.46  p=.04 | r=-.19  p=.40 | r=-.46  p=.04 | r=-.54  p=.01 | r=-.26  p=.26 |
| **DMN iFC** | r=.25  p=.27 | r=.35  p=.12 | r=.23  p=.32 | r=.35  p=.13 | r=.08  p=.70 |
| **DAN integrity** | r=-.33  p=.15 | r=.08  p=.72 | r=-.31  p=.17 | r=-.40  p=.07 | r=-.11  p=.62 |
| **DAN iFC** | r=-.21  p=.36 | r=-.06  p=.79 | r=-.36  p=.11 | r=-.27  p=.23 | r=-.24  p=.30 |
| **ASM integrity** | r=-.36  p=.11 | r=-.25  p=.28 | r=-.46  p=.04 | r=-.64  p=.002 | r=-.28  p=.22 |
| **ASM iFC** | r=.06  p=.79 | r=.33  p=.14 | r=.12  p=.60 | r=.21  p=.35 | r=.05  p=.83 |
| **LSD>**  **PCB DC** | r=.15  p=.46 | r=-.15  p=.46 | r=.16  p=.43 | r=-.08  p=.70 | r=-.12  p=.55 |
| **PCB>**  **LSD DC** | r=-.04  p=.81 | r=.09  p=.66 | r=-.00  p=.98 | r=.46  p=.01 | r=.15  p=.45 |
| **Associations between d-amphetamine-induced changes and 5D-ASC** | | | | | |
|  | **OSE** | **AIA** | **VUS** | **AUD** | **VIG** |
| **VIS integrity** | r=-.02  p=.91 | r=.21  p=.36 | r=-.04  p=.84 | r=-.13  p=.57 | r=.04  p=.86 |
| **VIS iFC** | r=-.16  p=.49 | r=.10  p=.65 | r=-.16  p=.48 | r=.33  p=.15 | r=-.21  p=.35 |
| **SAL integrity** | r=-.45  p=.04 | r=-.20  p=.38 | r=-.34  p=.14 | r=.14  p=.54 | r=-.35  p=.12 |
| **SAL iFC** | r=-.12  p=.59 | r=.06  p=.79 | r=-.04  p=.86 | r=.07  p=.74 | r=-.17  p=.46 |
| **FPN integrity** | r=-.30  p=.18 | r=-.05  p=.80 | r=-.19  p=.40 | r=-.00  p=.99 | r=-.47  p=.03 |
| **FPN iFC** | r=-.13  p=.57 | r=.26  p=.26 | r=.01  p=.94 | r=.32  p=.16 | r=-.23  p=.31 |
| **DMN integrity** | r=.03  p=.88 | r=.04  p=.86 | r=.13  p=.55 | r=-.04  p=.85 | r=.12  p=.61 |
| **DMN iFC** | r=-.07  p=.76 | r=.16  p=.49 | r=-.16  p=.48 | r=.22  p=.34 | r=-.11  p=.63 |
| **DAN integrity** | r=-.31  p=.18 | r=-.30  p=.19 | r=-.38  p=.09 | r=-.55  p=.01 | r=-.05  p=.81 |
| **DAN iFC** | r=-.22  p=.33 | r=-.20  p=.38 | r=-.29  p=.21 | r=.16  p=.49 | r=-.10  p=.65 |
| **ASM integrity** | r=-.34  p=.13 | r=-.12  p=.60 | r=-.40  p=.07 | r=.17  p=.45 | r=-.28  p=.21 |
| **ASM iFC** | r=-.13  p=.58 | r=.23  p=.32 | r=-.02  p=.93 | r=.48  p=.03 | r=-.28  p=.23 |
| **Amph.>**  **PCB DC** | r=-.11  p=.59 | r=-.03  p=.86 | r=-.11  p=.59 | r=-.31  p=.12 | r=.09  p=.64 |
| **PCB>**  **Amph. DC** | r=-.22  p=.27 | r=.05  p=.81 | r=-.03  p=.86 | r=-.16  p=.43 | r=.18  p=.36 |
| **Associations between MDMA-induced changes and 5D-ASC** | | | | | |
|  | **OSE** | **AIA** | **VUS** | **AUD** | **VIG** |
| **VIS integrity** | r=.42  p=.06 | r=.06  p=.80 | r=.25  p=.28 | r=.22  p=.34 | r=.05  p=.80 |
| **VIS iFC** | r=-.25  p=.28 | r=-.27  p=.23 | r=-.06  p=.77 | r=-.11  p=.63 | r=-.05  p=.81 |
| **SAL integrity** | r=.22  p=.34 | r=-.18  p=.43 | r=.05  p=.81 | r=.18  p=.43 | r=-.13  p=.58 |
| **SAL iFC** | r=.34  p=.13 | r=-.16  p=.48 | r=.13  p=.58 | r=.31  p=.18 | r=-.12  p=.61 |
| **FPN integrity** | r=.32  p=.16 | r=.15  p=.52 | r=.22  p=.33 | r=.20  p=.39 | r=.01  p=.96 |
| **FPN iFC** | r=-.21  p=.36 | r=-.27  p=.24 | r=-.07  p=.75 | r=-.13  p=.58 | r=-.06  p=.78 |
| **DMN integrity** | r=.28  p=.22 | r=-.03  p=.88 | r=.11  p=.62 | r=.23  p=.31 | r=-.11  p=.63 |
| **DMN iFC** | r=-.10  p=.64 | r=-.33  p=.15 | r=.00  p=.97 | r=-.03  p=.87 | r=.00  p=.98 |
| **DAN integrity** | r=.54  p=.01 | r=.02  p=.93 | r=.26  p=.26 | r=.17  p=.46 | r=.27  p=.24 |
| **DAN iFC** | - | - | - | - | - |
| **ASM integrity** | r=.23  p=.31 | r=-.38  p=.09 | r=.05  p=.80 | r=.26  p=.25 | r=-.39  p=.08 |
| **ASM iFC** | r=.02  p=.91 | r=.02  p=.90 | r=.07  p=.77 | r=.02  p=.92 | r=.02  p=.91 |
| **MDMA>**  **PCB DC** | r=-.28  p=.22 | r=-.28  p=.23 | r=-.09  p=.69 | r=-.23  p=.31 | r=.00  p=.99 |
| **PCB >**  **MDMA DC** | r=-.19  p=.40 | r=-.31  p=.17 | r=.04  p=.86 | r=.12  p=.59 | r=.09  p=.67 |

| **Present study** | | **Preller study^7^** | | **Tagliazucchi study^8^** | **Müller study^9^** |
| --- | --- | --- | --- | --- | --- |
| **Study Desing** | | | | | |
| - Cross-over design with 4 conditions: LSD, d-amphetamine, MDMA, & placebo - Substances delivered p.o. > 10 days apart | | - Cross-over design with 3 conditions: LSD, LSD + ketanserin, placebo - Substances delivered p.o. 2 weeks apart | | - Cross-over design with 2 conditions: LSD, placebo - Substances delivered i.v. 2 weeks apart | - Cross-over design with 2 conditions: LSD, placebo - Substances delivered p.o. >7 days apart |
| **Denoising Procedures** | | | | | |
| Without GSR | GSR | Without GSR | GSR | Without GSR | Without GSR |
| **Analytical Method** | | | | | |
| *Degree Centrality*   - Computed with C-PAC - Voxel-wise | | *Global Brain Connectivity*   - In-house scripts - Grayordinate-wise | | *Functional Connectivity Density*   - In-house scripts - ROI-wise | *Global Correlation*   - Computed with CONN - Voxel-wise |
| **LSD > PCB** | | | | | |
| Frontal, inferior temporal, sensory cortices, basal ganglia, & thalamus | Sensory cortices, basal ganglia, thalamus, insula, brainstem, inferior temporal, cerebellum | Cerebellum, basal ganglia, & thalamus, PCC/precuneus | Sensory cortices, motor & visual | Frontal, parietal, inferior temporal, visual, basal ganglia & thalamus | Basal ganglia & thalamus |
| **PCB > LSD** | | | | | |
| Visual, precuneus | Visual | Visual, insula, sensory and motor cortices | Precuneus, frontal, parietal, inferior temporal, basal ganglia, & thalamus | - | - |

## **Table S7:** Changes in global connectivity

# **Supplementary figures**

## **Figure S1:** Substance-induced changes in network integrity with GSR

Depicted are changes in network integrity (i.e., within-network connectivity) induced by LSD, d-amphetamine, and MDMA, compared to placebo with GSR. Repeated-measures ANOVAs were computed on dual regression-derived parameter estimates and corrected for multiple comparisons (P_FDR_<.05). * - depicts significant differences. Abbreviations: VIS – visual network, SAL – salience network, FPN – frontoparietal network, DMN – default mode network, ASM – auditory-sensorimotor network.

## **Figure S2:** Substance-induced changes in network segregation with GSR

Depicted are changes in network segregation (i.e., between-network connectivity) induced by LSD, d-amphetamine, and MDMA, compared to placebo with GSR. Repeated-measures ANOVAs were computed on Pearson correlation coefficients of dual regression-derived time series of pairs of networks, followed by r-to-z transformations, and corrected for multiple comparisons (P_FDR_<.05). * - depicts significant differences. Abbreviations: VIS – visual network, SAL – salience network, FPN – frontoparietal network, DMN – default mode network, ASM – auditory-sensorimotor network.

**Figure S3:** Substance-specific changes in seed-based iFC
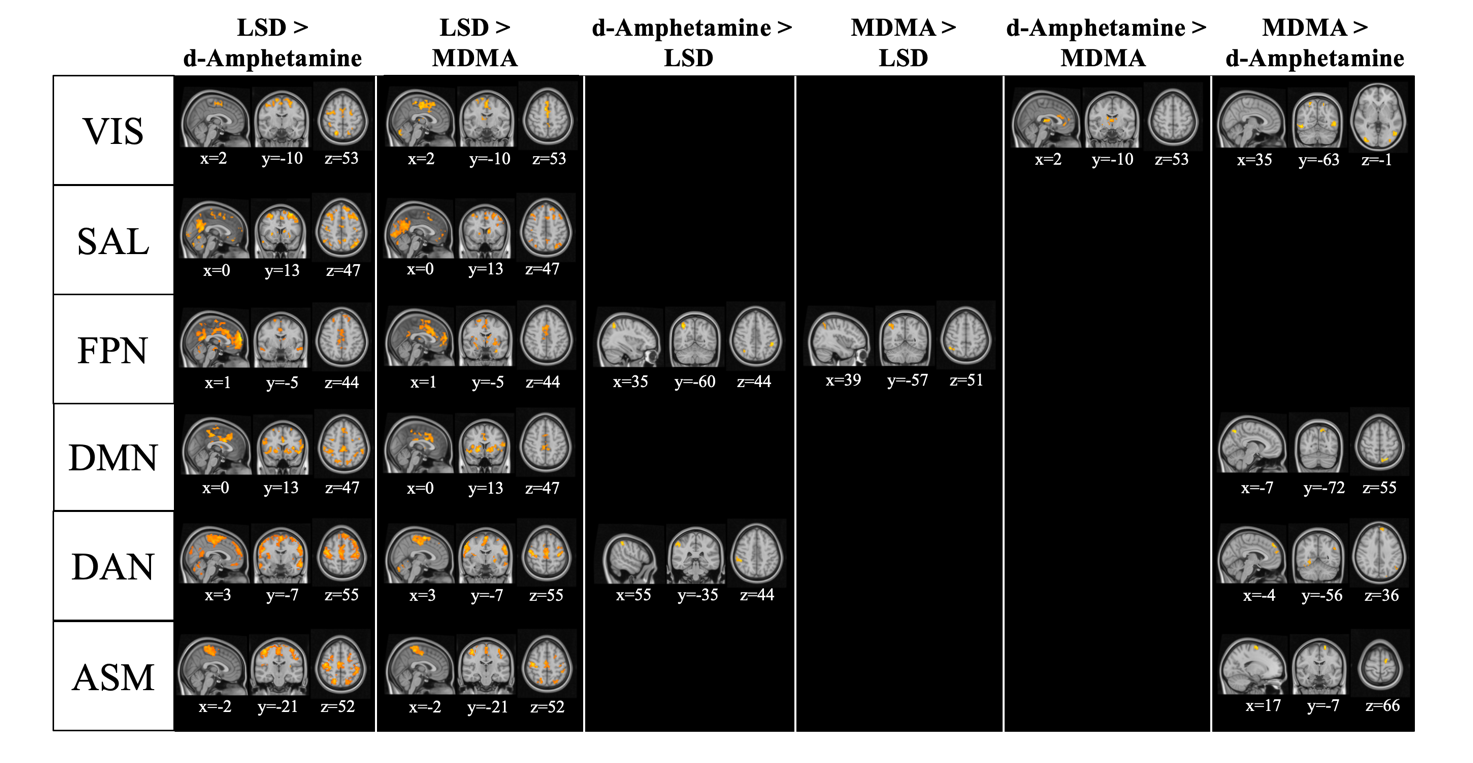


Depicted are voxel-wise repeated-measures analysis of variance (ANOVA) parametric maps reflecting contrasts between the active substances for RSN whole-brain connectivity. Only increased connectivity between the seed-network and other areas of the brain (shown in yellow/red) is shown. The analyses were computed in SPM12 (P<.001, cluster-level familywise error–corrected P<.05); x, y, and z indicate Montreal Neurological Institute coordinates.

## **Figure S4:** Substance-induced changes in seed-based iFC with GSR

Depicted are voxel-wise repeated-measures analysis of variance (ANOVA) parametric maps reflecting contrasts between active substances and placebo for RSN whole-brain connectivity with GSR. The substances mainly increased connectivity between the seed network and other areas of the brain (shown in yellow/red) but reduced connectivity within the seed network (shown in blue). The analyses were computed in SPM12 (P<.001, cluster-level familywise error–corrected P<.05); x, y, and z indicate Montreal Neurological Institute coordinates.

## **Figure S5:** Substance-specific changes in seed-based iFC with GSR

**

Depicted are voxel-wise repeated-measures analysis of variance (ANOVA) parametric maps reflecting contrasts between the active substances for RSN whole-brain connectivity. Only increased connectivity between the seed-network and other areas of the brain (shown in yellow/red) is shown. The analyses were computed in SPM12 (P<.001, cluster-level familywise error–corrected P<.05); x, y, and z indicate Montreal Neurological Institute coordinates.

## **Figure S6:** Global functional connectivity with GSR

**

Depicted are voxel-wise repeated-measures analysis of variance (ANOVA) parametric maps reflecting contrasts between active substances and placebo for global connectivity (Panel A) and between the active conditions only (Panel B) with GSR. The analyses were computed in SPM12 (P<.001, cluster-level familywise error–corrected P<.05); x, y, and z indicate Montreal Neurological Institute coordinates.

## **Figure S7:** The effects of GSR on within-condition differences in network segregation

The impact of GSR is illustrated on correlation coefficients between RSN pairs for each substance (LSD, d-amphetamine, MDMA) and placebo. The differences in correlation coefficients between data processed with and without GSR (wo. GSR - GSR) are displayed, with the largest effects observed for LSD, followed by d-amphetamine, MDMA, and placebo. Paired-t tests were conducted on Pearson correlation coefficients of dual regression-derived time series of pairs of networks, followed by r-to-z transformations, and corrected for multiple comparisons (P_FDR_<.05). Statistical control for heart rate, systolic and diastolic blood pressure, temperature, and mean framewise displacement did not affect these results, indicating that the differential impact of GSR is not merely due to distinct physiological effects or head motion. * - depicts significant differences. Abbreviations: VIS – visual network, SAL – salience network, FPN – frontoparietal network, DMN – default mode network, ASM – auditory-sensorimotor network.

# **Supplementary Code**

## **Computing network integration (bash script)**

# Set the used directories and subject file

*subject_dir=<path_to_subjects_directory>*

*output_dir=<path_to_output_directory>*

*subject_file=<file_ids_subject>*

# Set the studies networks, yeo_7 in our case

*YEO_rsn="NW01 NW02 NW03 NW04 NW05 NW06 NW07"*

# Generate a folder that outputs the mean PE values per network per subject per conditions (note that the values are ordered in the same way as the input files of dual regression)

*touch ${output_dir}/Intra_RSN${RSN}.txt*

*for RSN in `echo $YEO_rsn`*

*do*

*echo "$RSN"*

*for subject in $(cat ${subject_file}) #iterate for each subject file*

*do*

*echo "$ subject "*

*touch ${output_dir}/Integrity_${RSN}.txt*

# Please note you need the template for each network in the working directory

*fslmeants -i $subject_dir/${i}/${RSN}/dual_regression_map.nii.gz -m template_${RSN}.nii.gz -o ${ output_dir}/out.txt*

*cat ${output_dir}/out.txt >> ${output_dir}/Integrity_${RSN}.txt*

*rm ${output_dir}/out.txt*

*done*

*done*

## **Computing network segregation (R script)**

# Set the used directories, subject file, and studied networks’ name

*subject_dir=<path_to_subjects_directory>*

*output_dir=<path_to_output_directory>*

*subject_file=<file_ids_subject>*

*YEO_rsn="NW01 NW02 NW03 NW04 NW05 NW06 NW07"*

# Get the subjects IDs and the number

*subject_id = read.table(subject_file)*

*n = dim(subject_id)[1]*

# Compute the segregation for each subject (note that the values are ordered in the same way as the input files of dual regression)

*segregation = array(rep(NA, n*7*7), dim=c(n, 7, 7))*

*for (i in 1:n) {*

*subject = subject_id $V1[i]*

*print(subject)*

*all_RSN_ts = NULL*

*for (RSN in YEO_rsn) {*

*RSN_ts = read.table(paste0(subject_dir, "/", subject, "/”, RSN,*

*“/timeseries.txt"), header=F)*

*all_RSN_ts = rbind(all_RSN_ts, RSN_ts)*

*}*

*#* Compute the correlation and apply Fisher-z transform for each pair of networks

*RSN_ts_cor = cor(all_RSN_ts, all_RSN_ts)*

*RSN_ts_cor_z = atanh(RSN_ts_cor)*

# Save the segregation values

*segregation[i, , ] = RSN_ts_cor_z*

*}*

# Save the RDS file for network segregation

*write_rds(segregation, paste0(output_dir, “/segregation.rds")*

# **References**

1. American Psychiatric Association. *Diagnostic and Statistical Manual of Mental Disorders, Fifth Edition*. American Psychiatric Association: Arlington, VA, 2013.

2. Holze F, Vizeli P, Muller F, Ley L, Duerig R, Varghese N *et al.* Distinct acute effects of LSD, MDMA, and D-amphetamine in healthy subjects. *Neuropsychopharmacology* 2020; **45**(3)**:** 462-471.

3. McCulloch DE, Knudsen GM, Barrett FS, Doss MK, Carhart-Harris RL, Rosas FE *et al.* Psychedelic resting-state neuroimaging: A review and perspective on balancing replication and novel analyses. *Neurosci Biobehav Rev* 2022; **138:** 104689.

4. Griffiths RR, Johnson MW, Carducci MA, Umbricht A, Richards WA, Richards BD *et al.* Psilocybin produces substantial and sustained decreases in depression and anxiety in patients with life-threatening cancer: A randomized double-blind trial. *J Psychopharmacol* 2016; **30**(12)**:** 1181-1197.

5. Roseman L, Nutt DJ, Carhart-Harris RL. Quality of Acute Psychedelic Experience Predicts Therapeutic Efficacy of Psilocybin for Treatment-Resistant Depression. *Front Pharmacol* 2017; **8:** 974.

6. Goodwin GM. The psychedelic experience and treatment-resistant depression. *World Psychiatry* 2023; **22**(3)**:** 420-422.

7. Preller KH, Burt JB, Ji JL, Schleifer CH, Adkinson BD, Stampfli P *et al.* Changes in global and thalamic brain connectivity in LSD-induced altered states of consciousness are attributable to the 5-HT2A receptor. *Elife* 2018; **7**.

8. Tagliazucchi E, Roseman L, Kaelen M, Orban C, Muthukumaraswamy SD, Murphy K *et al.* Increased Global Functional Connectivity Correlates with LSD-Induced Ego Dissolution. *Curr Biol* 2016; **26**(8)**:** 1043-1050.

9. Muller F, Lenz C, Dolder P, Lang U, Schmidt A, Liechti M *et al.* Increased thalamic resting-state connectivity as a core driver of LSD-induced hallucinations. *Acta Psychiatr Scand* 2017; **136**(6)**:** 648-657.

10. Madsen MK, Stenbaek DS, Arvidsson A, Armand S, Marstrand-Joergensen MR, Johansen SS *et al.* Psilocybin-induced changes in brain network integrity and segregation correlate with plasma psilocin level and psychedelic experience. *Eur Neuropsychopharmacol* 2021; **50:** 121-132.

11. Timmermann C, Roseman L, Haridas S, Rosas FE, Luan L, Kettner H *et al.* Human brain effects of DMT assessed via EEG-fMRI. *Proc Natl Acad Sci U S A* 2023; **120**(13)**:** e2218949120.

12. Murphy K, Fox MD. Towards a consensus regarding global signal regression for resting state functional connectivity MRI. *Neuroimage* 2017; **154:** 169-173.

13. Yang GJ, Murray JD, Repovs G, Cole MW, Savic A, Glasser MF *et al.* Altered global brain signal in schizophrenia. *Proc Natl Acad Sci U S A* 2014; **111**(20)**:** 7438-7443.

14. Tanabe S, Huang Z, Zhang J, Chen Y, Fogel S, Doyon J *et al.* Altered Global Brain Signal during Physiologic, Pharmacologic, and Pathologic States of Unconsciousness in Humans and Rats. *Anesthesiology* 2020; **132**(6)**:** 1392-1406.
